# Dropout from psychological treatment for borderline personality disorder: a multilevel survival meta-analysis

**DOI:** 10.1017/S0033291722003634

**Published:** 2023-02

**Authors:** Arnoud Arntz, Kyra Mensink, Wouter R. Cox, Rogier E. J. Verhoef, Arnold A. P. van Emmerik, Sophie A. Rameckers, Theresa Badenbach, Raoul P. P. P. Grasman

**Affiliations:** 1 Department of Clinical Psychology, University of Amsterdam, Amsterdam, The Netherlands; 2 Department of Psychological Methods, University of Amsterdam, Amsterdam, The Netherlands

**Keywords:** Borderline personality disorder, dropout, meta-analysis, psychotherapy, treatment retention

## Abstract

**Background:**

Dropout from psychotherapy for borderline personality disorder (BPD) is a notorious problem. We investigated whether treatment, treatment format, treatment setting, substance use exclusion criteria, proportion males, mean age, country, and other variables influenced dropout.

**Methods:**

From Pubmed, Embase, Cochrane, Psycinfo and other sources, 111 studies (159 treatment arms, *N* = 9100) of psychotherapy for non-forensic adult patients with BPD were included. Dropout per quarter during one year of treatment was analyzed on participant level with multilevel survival analysis, to deal with multiple predictors, nonconstant dropout chance over time, and censored data. Multiple imputation was used to estimate quarter of drop-out if unreported. Sensitivity analyses were done by excluding DBT-arms with deviating push-out rules.

**Results:**

Dropout was highest in the first quarter of treatment. Schema therapy had the lowest dropout overall, and mentalization-based treatment in the first two quarters. Community treatment by experts had the highest dropout. Moreover, individual therapy had lowest dropout, group therapy highest, with combined formats in-between. Other variables such as age or substance-use exclusion criteria were not associated with dropout.

**Conclusion:**

The findings do not support claims that all treatments are equal, and indicate that efforts to reduce dropout should focus on early stages of treatment and on group treatment.

## Introduction

Psychological treatment of borderline personality disorder (BPD) is usually considered as being highly complex, with treatment discontinuation before a significant improvement has been reached as one of the most challenging problems. Early reports documented treatment dropout rates higher than 50% within 6 months of traditional psychotherapy (Gunderson et al., 1989; Skodol, Buckley, & Charles, 1983; Waldinger & Gunderson, 1984). The high dropout rate among patients with BPD is generally viewed as related to their complex psychopathology, including impulsivity, anger problems, and difficulties in establishing trusting relationships. High dropout risk is problematic given the high levels of disfunctioning, high suicide risk, and high societal costs associated with BPD (Lieb, Zanarini, Schmahl, Linehan, & Bohus, 2004; van Asselt, Dirksen, Arntz, & Severens, 2007; Wagner et al., 2022). It is also demotivating for therapists, who have to invest a lot in the treatment of difficult patients, and are confronted with many patients that end treatment prematurely. Moreover, premature treatment discontinuation constitutes a threat for the cost-effectiveness of the intensive and costly interventions for BPD, that are often available only for a limited number of BPD-patients. Understandably, one of the aims of specialized psychotherapies such as dialectical behavior therapy (DBT), transference-focused psychotherapy (TFP), mentalization-based treatment (MBT) and schema therapy (ST) (the ‘big-four’), that were developed since the late eighties of the previous century, was therefore the reduction of treatment dropout.

Attempts to create comprehensive quantitative overviews of dropout rates of different treatments of BPD (e.g. Barnicot, Katsakou, Marougka, & Priebe, 2011) have been limited by a number of factors. First, treatments and studies of treatments vary widely in the time period they cover, making a simple meta-analytic approach of risk of dropout without taking the duration of treatment into account senseless. Second, the standard meta-analytic approach to only include randomized clinical trials (RCTs) severely limits the comparison possibilities between treatment approaches, as many have not been directly compared. Third, many studies that have been published are based on a non-RCT design, and disregarding them, while methodologically sound from one point of view, seriously limits the comprehensiveness in terms of the available evidence and the type of treatments that can be studied. Fourth, BPD treatments have been studied in different settings (inpatient, day treatment, outpatient) and in different formats (individual, group, and combined) which raises the question how these variables are related to treatment retention. Fifth, meta-regression offers the opportunity for a multivariate analysis of putative factors predicting treatment dropout.

Various factors on different levels have been suggested to relate to treatment dropout. First, patient characteristics, such as male gender and younger age (Arntz, Stupar-Rutenfrans, Bloo, van Dyck, & Spinhoven, 2015; Crawford et al., 2009; Edlund et al., 2002; McMurran, Huband, & Overton, 2010). Second, treatment characteristics, such as treatment model, format (i.e. group, individual, and combined group-individual), and setting (outpatient, day-treatment and inpatient). As to treatment models, for the ‘big-four’ treatments (DBT, ST, MBT, TFP) superior treatment retention has been claimed. As to format, group therapy tends to have higher dropout rates than individual therapy, which might relate to practical issues (less agenda flexibility) as well as psychological factors (e.g. groups might be threatening for patients) (MacNair & Corazzini, 1994; Yalom, 1966). The present authors are not aware of any claims as to setting, but it seems an important factor to investigate. Third, there are socio-economic factors that might be related to treatment retention (e.g. the general difference between European public health care *v.* the limited availability of mental health care for poor people in the US might influence dropout from studies; Edlund *et al*., 2002; Gaglia, Essletzbichler, Barnicot, Bhatti, & Priebe, 2013; McMain *et al*., 2009; Priebe *et al*., 2012).^†^^1^ Lastly, study design factors might be related to dropout. RCTs have a higher methodological status than open trials, and perhaps the latter report lower dropout rates than RCTs, suggesting biases in reporting dropout rates in open trials. Moreover, studies use different exclusion criteria and the in- *v.* exclusion of different levels of substance use disorders might be especially important for treatment retention, with more lenient criteria perhaps being associated with higher dropout.^2^

The aim of the present meta-analysis was therefore to study dropout from psychological treatments for BPD, taking into account as much data as possible, including data from non-RCTs, investigating various factors that might influence treatment dropout. We chose survival analysis as this approach is suitable for analyzing dropout data from observational periods of varying lengths, while allowing the use of multiple covariates, and we chose a multilevel approach so that studies could be combined in one analysis and different predictors could be tested. More specifically, we aimed to address the following questions:

1. How do different psychological treatments compare in terms of treatment retention?
2. Is setting (inpatient, day-treatment, outpatient) associated with treatment retention?
3. Is treatment format (individual, group, combined individual-group) associated with treatment retention?
4. Is gender composition of the study sample associated with dropout?
5. Is the sample's mean age associated with treatment retention?
6. Is study quality associated with treatment retention?
7. Are methodological aspects of studies, like trial design (RCT, non-RCT, open trial), and distinction *v.* non-distinction of treatment and study dropouts, associated with dropout?
8. Is country where the study was conducted related to treatment retention?
9. Does type of exclusion of substance use disorders relate to treatment retention?
10. Has treatment retention improved over the years that we study BPD treatments?

Initially, we also aimed to investigate the association of suicidality, comorbidity, educational level, and unemployment with treatment retention. However, too many studies did not present (suitable) data on these variables, so that we had to disregard this aim.

## Methods

### Guidelines for meta analyses

For this meta-analysis, we followed the PRISMA Guidelines (Moher, Liberati, Tetzlaff, & Altman, 2009) and the American Psychological Association MARS Guidelines (APA, 2008). However, we did not preregister the meta-analysis.

### Identification and selection of studies

A database search was done in Pubmed, Embase, Cochrane, and Psycinfo, on 21 June 2013; and was repeated on 4 February 2015 and January 31 and 15 June 2022. Appendix A (appendices can be found in the supplementary material) provides the search terms. In total, 2997 records were retrieved. In addition, reference lists of reviews, meta-analyses, and other manuscripts were checked, and one submitted ms. was obtained with permission of the authors, which yielded another 880 records. The following criteria were used by three independent judges (from PC, AvE, AA, RV) to select studies for inclusion in the meta-analysis. In case of disagreement, a decision was reached through consensus.

Inclusion criteria:

1. a study of psychological treatment for BPD: RCT, open trial, case series, cohort study.
2. adult patients (age ⩾18) with primary diagnosis of BPD according to DSM-III, DSM-III-R or DSM-IV (-Tr) criteria.

Exclusion criteria:

1. ‘double diagnoses’: study focuses exclusively on a specific combination of two diagnoses, e.g. BPD and eating disorder; BPD and opioid dependence. The reason was that such studies have biased sampling from the BPD population; and that treatments are modified to the double diagnosis.
2. single case studies: in contrast to consecutive case series studies, there is little guarantee that reporting is not biased (i.e. the case was only reported if a success).
3. mixed PD – samples; unless separate statistics on the BPD subsample are given. A tolerance of 10% was allowed: at least 90% had to meet full BPD diagnosis. Thus a study was excluded if more than 10% did not meet full BPD-diagnosis (in case of mixed samples). Authors of studies published after 2000 were asked for statistics of the BPD-subsample.
4. treatments that consist of subsets of techniques or modules which are clearly not intended to be complete treatments: i.e. incomplete parts of treatments. However, tests of protocols intended to be complete treatments but missing usual ingredients of the protocol are included and are marked as such (e.g. ‘reduced DBT’, also labeled ‘DBT-min’, for DBT treatments without a specific ingredient).
5. treatment modules that are explicitly additions to treatments, i.e. psycho-education; courses; specific skills training, as these are not complete treatments (for example: STEPPS; psycho-education). One study tested a complete STEPPS treatment by adding protocolized individual sessions to group, and hence was included.
6. forensic populations, as these require specific forms of treatment, and effects and dropout are difficult to generalize outside the forensic context.
7. no dropout data reported, as without data on dropout, the study could not be included in the analysis (in case there were unclarities, authors were contacted if published after 2000).

Note that studies might have treatments that meet selection criteria as well as treatments that do not meet them. E.g. some studies on training add-on's had a TAU comparison condition. In such cases the TAU condition data was included, if it passed the selection criteria.

**Fig. 1.**
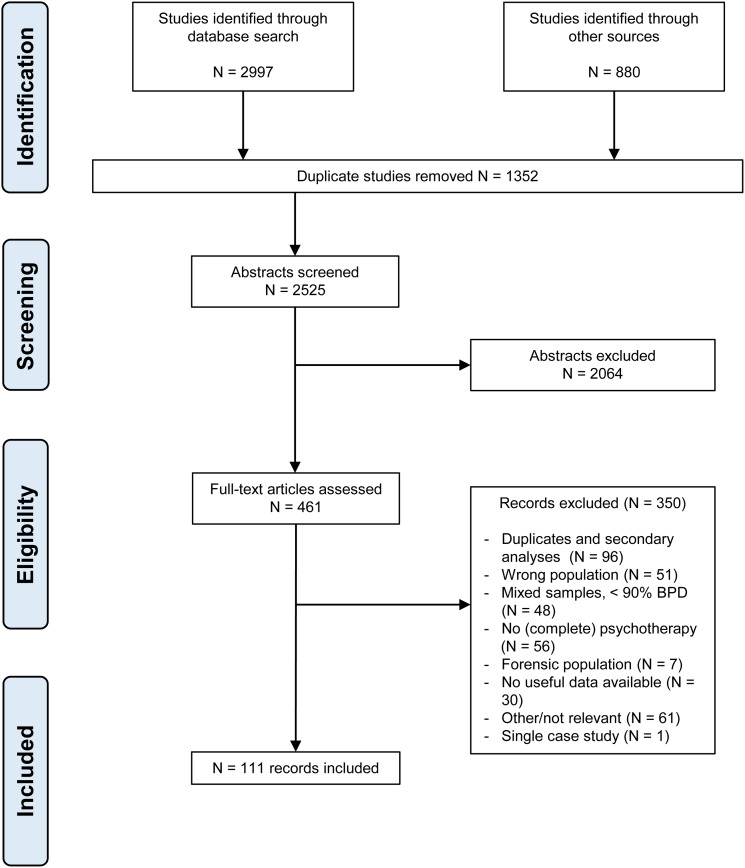
Flowchart of study selection.

If an English abstract was available and survived the initial screening, language was not an eligibility criterion, and non-English, non-Dutch or non-German papers were translated into English for further scrutiny. Figure 1 shows the flowchart of the study selection. Appendix B gives an overview of the characteristics of the studies included, appendix C their references. As a result of the criterion that diagnoses should be based on DSM-III or later editions, the earliest included study was published in 1990.

### Treatment definition

We initially classified treatments into 17 categories (n's refer to the total sample sizes): DBT (*n* = 3916); reduced DBT (*n* = 716); ST (*n* = 539); MBT (*n* = 448); TFP (*n* = 163); cognitive-behavioral therapy (CBT; *n* = 258); psychodynamic therapy (PsyDyn; *n* = 723); cognitive-analytic therapy (CAT; *n* = 61); interpersonal therapy (IPT; *n* = 60); client-centered therapy (CCT; *n* = 44); structural clinical management (SCM; *n* = 63); general psychiatric management (GPM; *n* = 90); therapeutic community (TherCom; *n* = 78); community treatment by experts (CTBE; *n* = 101); treatment-as-usual (TAU; *n* = 728); Dynamic Deconstructive Psychotherapy (DDP; *n* = 42); and ‘mixed’ (*n* = 1070), treatments combining different approaches such as CBT with psychodynamic, or DBT with TFP, as well as treatment arms consisting of individually allocated specialized treatments. Following the developer of DBT (Linehan et al., 2015), a DBT-treatment was classified as full DBT if it included four standard DBT components [group skills training, individual coaching, outside session telephone crisis support, therapist consultation (in case of inpatient DBT, outside session crisis support was assumed)]. If one component was not present, the treatment was classified as reduced DBT. Some studies deliberately tested reduced DBT.

We next reduced the number of treatment categories by collapsing specified treatment categories with *n* < 100 together with the mixed category into a ‘specified others’ category (*N* = 1432). The ‘specified others’ category thus consisted of psychotherapies that had at least some adjustment to BPD, but individually had a *n* < 100. CTBE was distinguished from TAU as it is viewed as an optimized variant of TAU, and thus constitutes a more stringent comparison condition than TAU (e.g. Linehan et al., 2006).

### Coding of methodological quality (risk of bias) of studies

Included studies were assessed for risk of bias by evaluating nine design criteria. These criteria were based on Cuijpers, van Straten, Bohlmeijer, Hollon, and Andersson (2010) and slightly modified to accommodate the BPD treatment outcome literature, including non-RCTs. Specifically, we evaluated for each treatment arm whether: (1) the BPD diagnosis was made using semi-structured diagnostic interviews such as the SCID-II (First, Gibbon, Spitzer, Williams, & Benjamin, 1997) [0 = no or unknown, 1 = yes, but with inadequate or unknown inter-rater reliability (IRR), 2 = yes, with adequate IRR]; (2) a treatment manual was used (0 = no or unknown, 1 = yes, but treatment manual is unpublished, 2 = yes, with published treatment manual); (3) therapists were trained either specifically for the study or in a general training (0 = no or unknown, 1 = no or unknown, but therapists are clearly experts, 2 = yes); (4) treatment integrity was checked (0 = no or unknown, 1 = yes, by supervision, 2 = yes by independent raters); (5) the study was randomized (0 = no or unknown, 1 = yes, but randomization was partly violated, 2 = yes); (6) if applicable, whether randomization was independent and adequately concealed (0 = no or unknown, 1 = either independent or adequately concealed, 2 = both independent and adequately concealed); (7) if applicable, whether assessment interviews were conducted by independent or blind assessors (0 = no or unknown, 1 = yes, independent but not blind, 2 = yes, blind); and (8) whether and how dropout was reported (0 = no, 1 = yes, but no distinction between types of dropout, 2 = yes, with adequate distinction between types of dropout). If a study investigated DBT, ST, TFP or MBT, criterion 2 was coded as 2 (i.e. using a published treatment manual).

Following calibration exercises on a subset of included studies, the remaining studies were independently rated on these criteria by different pairs from a total of five coders. Interrater agreement of the initial ratings was assessed using two-way mixed, absolute agreement, average-measures intra-class correlations (ICCs). The ICC per item ranged from 0.86 to 0.97, with a mean and median of 0.90. The ratings were summarized into a mean score (range 0–2; internal consistency 0.69, ICC 0.95) for each study's arm (see Appendix B). Note that treatment arms per study could get different ratings, e.g. in RCTs that compared a manualized therapy to a non-manualized TAU. The study arm's quality score was used as covariate in the analyses.

### Coding of dropout and other characteristics

Two raters (from KM, WC, SR, TB) independently coded treatment characteristics and dropout per quarter. In case of disagreement, the issue was resolved through discussion. In several cases authors were contacted by email to clarify issues. Dropout per quarter was derived from the article's text (methods, results, discussion sections), flow diagram, or survival curve. When quarter of dropout was not clear from the report, authors of studies published after 2000 were emailed but not all were able or willing to provide details (Appendix D). Most studies did not distinguish between ‘dropouts’ and ‘pushouts’, the first being based on patients' decisions, the latter on therapists' decisions or protocol rule. We therefore couldn't distinguish between these types of dropout. Moreover, most studies did not report details about reasons for dropout, thus for these studies we took all treatment dropouts into account. However, when reasons clearly not related to treatment were reported, these cases were not considered as treatment dropout. Type of exclusion of substance use was coded as no exclusion, clinical detox needed excluded, substance dependence excluded, substance abuse excluded, and unclear. Country of the study was classified in four categories: Europe, USA, Australia/Canada/New Zealand, and emerging (China/Iran/Mexico).

### Statistical analysis

A multilevel survival analysis was used to analyze treatment discontinuation over time, using a random effects approach by adding study as random factor to the model. This method allows for testing of multiple predictors whilst controlling for other variables, and for distinguishing between individual cases and studies on separate levels. Moreover, the method can handle censored data, which is necessary as study length varies, and cases ‘disappear’ when they stop treatment prematurely. Study served as random factor in the analysis, representing random variation between studies. This approach models the included studies as a random sample from a population of studies (hence, from the BPD population, treatment centers, therapists) and allows generalization of the findings (e.g. Hedges & Vevea, 1998). The statistical test of this random factor is reported in the results section. Although multilevel continuous time survival methods have been developed, they are not currently accessible through widely available software. Furthermore, they tend to suffer from numerical instability (Eager & Roy, 2017), and require exact dropout times for each patient which is not always available. Therefore we used multilevel survival analysis with quarter as time period. As survival chance might differ per quarter, quarter was entered as factor in the analysis. Interval censored survival analysis of Generalized Linear Mixed Models of SPSS version 28 was used (International Business Machines, 2021), which uses a binomial distribution with a complementary log-log link. We used Restricted Maximum Likelihood estimation with the Satterthwaite method for defining degrees of freedom in the *t* tests of the fixed effects coefficients (Luke, 2017).

For the analyses the numbers of cases (dis)continuing treatment in the specific quarter were reconstructed on the basis of the reports of the studies. In case the study period was not equal to a complete number of quarters (e.g. study length was 2.5 quarters), we estimated the dropout for the last quarter of the study, assuming constant survival chance for that quarter. For 34 studies (45 treatment arms) dropout was reported, but not in enough detail to reconstruct dropout per quarter [for seven of these studies only a part of the dropout development had to be estimated (in 11 arms)]. For these we estimated dropout per quarter based on the general development of treatment dropout based on studies reporting dropout per quarter. Note that the reported total dropout formed the basis of each estimation, i.e. the estimated dropouts per quarter sum up to the total number of dropouts reported. Available data showed clear evidence for a smaller retention rate in the first quarter than in later quarters, with a gradual increase in retention in later quarters. This time-dependence was best described by a logistic time model and this model was therefore used to estimate dropout per quarter which was subsequently used in the multiple imputation (MI) model (Appendix E). Appendix D gives an overview of treatment dropout per quarter per study arm, including the estimated dropout per quarter.

Because the estimation of dropout per quarter for studies not reporting this detail underestimates variance in retention rate per quarter, we used a MI strategy to deal with this. Twenty datasets were created with, for studies with incomplete details, random varying dropout numbers based on a binomial distribution of the numbers showing a particular dropout pattern, with the numbers of treatment completers as well as the total dropout number held constant. Appendix F provides details of this MI procedure. For studies not reporting mean age and/or proportion male participants, these variables were also estimated by MI. For model selection, the resulting 20 datasets were analyzed in one GLMM survival analysis, with for dropout-pattern of each set a weight of *n*/20, with *n* = number of participants of the pattern, so that the total sample size of the 20 combined sets was equal to the observed sample size.^3^

For model selection, we first entered all covariates as main effects in the fixed part and then applied stepwise deletion of covariates with significance level >0.05. For the remaining covariates it was next tested whether the interaction with quarter was significant. Both the full model with all main effects and the final models (with significant main effects, and with significant interactions with quarter) are presented. Note that because of model selection, the *p* values are indicative. For the final models, significant effects of categorical variables were further tested by deviation contrasts, which test the difference of a category with the overall mean. This way, the number of comparisons is not too large (e.g. with 10 treatment models, there are 10 deviation contrasts compared to 45 pairwise comparisons). Deviation contrasts test what is generally most relevant when large numbers of conditions are investigated, i.e. which conditions differ from the general picture. For the final step of testing the selected model, we used a conventional MI procedure with Rubin's rule for estimating means, their s.e.'s, and the deviation contrasts and their *t* tests and *p* values (this procedure turned out to be more conservative than the procedure used for model selection, which ensures that not too optimistic levels of significance are reported).

The following variables were initially entered as covariates: quarter, study design (RCT, open trial, nonrandomized controlled), dropout type (treatment dropout *v.* no distinction made between treatment and study dropout), medication policy (prescribed *v.* nonprescribed medication), treatment model, treatment format (individual, group, combined), treatment offered in addition to TAU (yes/no), setting (inpatient, outpatient, day treatment), dropout imputed in case of insufficient details in study report (yes/no), proportion males, mean age, methodological quality, publication year, exclusion of substance disorders, and country group (Europe, USA, Australia/Canada/New Zealand, emerging).

Survival curves were constructed from the estimated means from the fixed part, controlled for the indicated covariates. The period of investigation was limited to the first year, as very few studies had a study length beyond one year, which led to estimation problems in the statistical analysis of longer periods.

The random effect *I*^2^ of study was derived from the random effect estimate: *I*^2^ = random effect/(1 + random effect) × 100%. Egger's test was used to test whether precision was associated with treatment retention by adding 1/√*N* as covariate to the fixed part of the final model, with N = sample size of the study arm. Lastly, funnel plots were constructed by plotting residuals of the final analysis against study precision (i.e. the s.e. of the observed treatment retention at quarter *i*, with s.e.*_i_* = √(*p_i_* (1–*p_i_*)/*N_i_*), with *p_i_* = retention proportion in quarter *i*, and *N_i_* = sample size of quarter *i*. In case of low dropout, <17%, the Agresti–Coull approximation was used by defining *p_i_* = (*n_i_* + 2)/(*N_i_* + 4) (Agresti & Coull, 1998).

In addition to the prespecified analysis described above, a sensitivity analysis was done excluding DBT treatment arms that used different pushout rules than the DBT protocol prescribes.

### Selected studies

There were 111 studies (159 treatment arms, total *N* = 9100) that met inclusion criteria and reported treatment dropout data. Table 1 gives an overview of these studies; appendix B shows the characteristics per study arm. In short, sample size per arm varied from *N* = 5 to *N* = 1423 (mean *N* = 57.2, median *N* = 33); 54 studies investigated DBT [*N* = 4632 (*N* = 3916 full DBT; *N* = 716 reduced DBT); 11 studies investigated solely (a) reduced form(s) of DBT, two both full and reduced forms of DBT]; 12 ST (*N* = 539); 5 TFP (*N* = 163); 8 MBT (*N* = 448); 8 CBT (*N* = 258); 25 TAU (*N* = 728); 11 psychodynamic psychotherapy (*N* = 723); 3 CAT (*N* = 61); 3 IPT (*N* = 60); 2 CCT (*N* = 44); 1 SCM (*N* = 63); 1 GPM (*N* = 90); 8 mixed approaches, using combinations of models (*N* = 824) or a range of specified therapies (1; *N* = 246); 2 CTBE (*N* = 101); 2 Therapeutic Communities (*N* = 78); 2 DDP (*N* = 42). Study-arms varied in length from one quarter (32, *N* = 3407) up to 3 years (3, *N* = 94), with 32 spanning 2 quarters (*N* = 1648), 10 3 quarters (*N* = 269), 58 one year (*N* = 2251), 27 longer than one year (*N* = 1525). Over all studies, the mean of the study's mean age of patients was 31.02 (s.d. 4.31; median 31.30; range 20.40–40.10), seven studies did not report age (for the analysis, missings were handled by MI). The mean proportion male patients was 0.150 (s.d. 0.130; median 0.138; range 0–0.560), eight studies did not report gender composition (for the analysis, missings were handled by MI). Year of publication varied from 1990 to 2022 (one study was submitted and labeled as 2015), with mean 2011 and median 2011. Most studies investigated outpatient treatment (79% of arms). Combined individual-group therapies were most often investigated (57% of arms). RCTs were the most common (52.8% of arms). Most studies distinguished between treatment and study dropout (80.5% of arms). Most studies used an intent-to-treat approach (57.2% of arms), though a completers analysis only was not uncommon. Seven studies investigated psychotherapy delivered in addition to TAU (5% of arms), and five studies had a prescribed medication policy. The majority of studies (and participants) came from Europe (*N* = 6458), followed by USA (1544), Australia/Canada/New Zealand (*N* = 757), and emerging countries (*N* = 341) (Table 1). As to substance abuse related exclusion, 62 study-arms did not report exclusion of substance related disorders (*N* = 2985), 33 excluded only when a clinical detox was necessary (*N* = 3202), 40 excluded substance dependence (*N* = 2170), 20 substance abuse (*N* = 623), and 4 (*N* = 120) were unclear about this. All these variables were used as covariates.

**Table 1.** Number of studies and sample sizes by treatment and study characteristics

	# studies		*N*			# studies		*N*	# studies	*N*		
Setting^a^	Outpatient					Day-treatment			Inpatient			
	83		4938			15		1158	17	3004		
Format^a^	Individual + Group					Individual			Group			
	74		6595			43		1652	13	853		
Study design	Open Trial					RCT			Non-randomized controlled			
	58		5008			44		3397	9	695		
Add-on to TAU^a^	Not added to TAU					Added to TAU						
	110		8853			7		247				
Medication	Not prescribed					Prescribed						
	106		8946			5		154				
Dropout type	Treatment dropout distinguished from Study dropout					Study and treatment dropout not distinguished						
	90		7775			21		1325				
Dropout per Quarter^b^	Completely reported					Partially reported			Not reported			
	87		7232			7		483	20	1385		
Country group	Europe			USA			Australia/Canada/NewZealand			Emerging		
	48	4781		27		1089	6		418	418	341	

a Note that some studies investigated multiple settings/formats/models. Hence, #studies >111 in these rows.

b Note that some studies had complete dropout reports for one arm, but not or partially reported for another arm. Hence, #studies >111 in this row.

## Results

### All studies included

The upper part of Table 2 presents the results of the fixed part of the initial model with all main effects. The middle part presents the results after stepwise deletion of predictors with *p* > 0.05, the lower part results with significant interactions added.

**Table 2.** *F*-tests of predictors of treatment retention of the fixed part of the initial and final models

Model	*F*	df1	df2	*p*
Initial model				
Corrected model	7.041	33	176	<0.001
Year of publication	1.940	1	69	0.17
Methodological quality of study's arm	<0.001	1	116	0.99
Trial type	0.135	2	74	0.87
Dropout type distinguished	0.604	1	66	0.44
TAU added to treatment	0.727	1	601	0.39
Dropout per quarter to be imputed*	**4.723**	**1**	**129**	**0.032**
Setting (inpatient, day treatment, outpatient)	1.485	2	256	0.23
Treatment Format (Individual, Group, Combined)	2.723	2	1397	0.066
Medicine prescribed by study	0.774	1	81	0.38
Substance use exclusion	0.539	4	70	0.71
Country group (Europe, USA, Aus/Can/NZ, emerging)	0.140	3	67	0.94
Mean age of study's arm	0.582	1	486	0.45
Proportion males of study's arm	0.159	1	346	0.69
Treatment category*	**5.952**	**9**	**955**	**<0.001**
Quarter*	**50.305**	**3**	**23 546**	**<0.001**
Intermediate model (Main effects after backwards deletion)				
Corrected model	15.281	15	1230	<0.001
Dropout per quarter to be imputed*	**4.868**	**1**	**128**	**0.029**
Treatment format (Individual, Group, Combined)*	**3.607**	**2**	**988**	**0.027**
Treatment category*	**6.533**	**9**	**1655**	**<0.001**
Quarter*	**51.505**	**3**	**23 564**	**<0.001**
Final main and interaction effects model				
Corrected model	7.308	42	5000	<0.001
Dropout per quarter to be imputed*	**4.867**	**1**	**125**	**0.029**
Treatment format (Individual, Group, Combined)*	**3.954**	**2**	**1011**	**0.019**
Treatment category*	**5.353**	**9**	**2120**	**<0.001**
Quarter*	**37.623**	**3**	**23 537**	**<0.001**
Treatment category × quarter*	**2.849**	**27**	**23 537**	**<0.001**
Egger's test (1/√(*N*)*	**5.996**	**1**	**158**	**0.015**

*Significant effects are printed in **bold.**

#### Initial model

Study as random factor was significant, *z* = 4.825, *p* < 0.001 (*I*^2^ = 100*.135/1.135 = 11.9%). In the full model, quarter was significant, with growing treatment retention over time. Using imputation for number of dropouts per quarter was associated with less retention (*β* = −0.171, *p* = 0.032). Treatment category was significant (details: see final model). The trend in format was related to group having lower treatment retention than individual and combined formats.

#### Intermediate model

Only the main effects of quarter, dropout per quarter imputation, format, and treatment category survived the backward deletion (Table 2).

#### Final model

**Fig. 2.**
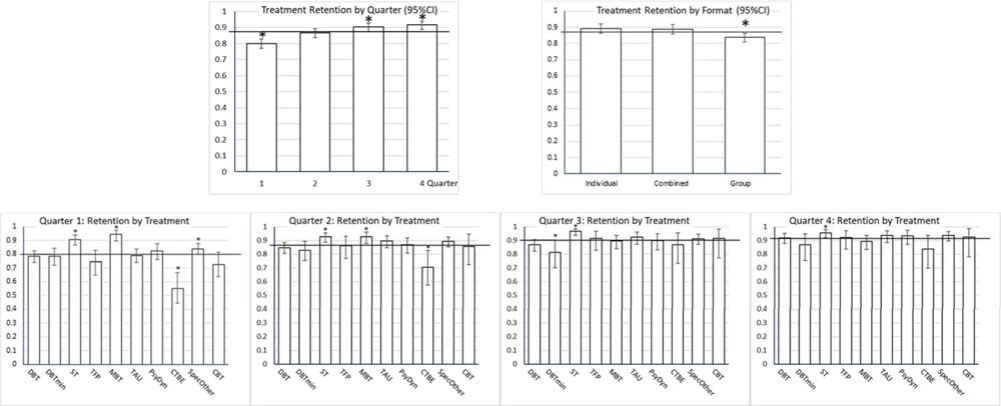
Treatment retention proportion per quarter (with 95%CI) as estimated in the complete dataset. The horizontal line is the average treatment retention, to which the estimated effects are compared (deviation contrasts). Significant effects (*p* < 0.05) indicated by *. Upper left panel: treatment retention by quarter, showing increasing retention with time. Upper right panel: treatment retention by treatment format, showing significantly less retention in group treatment. Lower panels: treatment retention by treatment types and quarter. In all quarters, ST had significantly higher treatment retention than average. In quarters 1 and 2 MBT had significantly higher retention, CTBE significantly less, than average. Reduced DBT (DBTmin) had significantly less retention in quarter 3.

Of the interactions only that between treatment category and quarter was significant. The lower part of Table 2 shows the final model. The random effect of study was significant, *z* = 5.219, *p* < 0.001 (*I*^2^ = 100*.110/1.110 = 9.9%). Egger's test was significant, with lower precision associated with less treatment retention. However, only 3.7% of the variance was associated with imprecision (*r*^2^ = *F*/(*F* + df)). Follow-up contrasts are reported in Table 3. Quarter was significant, with the lowest retention chance in the first quarter, and gradual increase in retention in later quarters. (Fig. 2 shows the estimated retention chances for these effects).

**Table 3.** Nominal predictors: Retention chances and follow-up contrasts of the final model (MI on complete study set)

		Transformed scale				Estimated survival chance per quarter		
Predictor		Pooled mean	Rubin's s.e.	Deviation contrast		Pooled mean	95% CI	
				*t*	*p*		Lower	Upper
Dropout per quarter imputed*								
No*		**0.803**	**0.049**	**2.101**	**0.038**	**0.893**	**0.869**	**0.914**
Yes*		**0.657**	**0.064**	**−2.101**	**0.038**	**0.855**	**0.818**	**0.887**
Treatment format*								
Individual		0.807	0.054	1.706	0.09	0.894	0.867	0.917
Combined individual-group		0.784	0.052	1.602	0.11	0.888	0.862	0.912
Group*		**0.598**	**0.077**	**−2.724**	**0.007**	**0.838**	**0.791**	**0.879**
Quarter*								
Quarter 1*		**0.475**	**0.048**	**−9.244**	**<0.001**	**0.800**	**0.769**	**0.829**
Quarter 2		0.696	0.054	−1.020	0.31	0.865	0.835	0.893
Quarter 3*		**0.843**	**0.060**	**3.095**	**0.002**	**0.902**	**0.873**	**0.927**
Quarter 4*		**0.905**	**0.063**	**4.581**	**<0.001**	**0.916**	**0.888**	**0.939**
Treatment category*								
DBT		0.670	0.060	−1.084	0.28	0.858	0.824	0.889
DBTreduced		0.553	0.095	−1.930	0.054	0.824	0.764	0.877
ST*		**1.049**	**0.071**	**5.000**	**<0.001**	**0.942**	**0.916**	**0.962**
TFP		0.705	0.104	−0.280	0.78	0.868	0.808	0.916
MBT*		**0.908**	**0.081**	**2.580**	**0.010**	**0.916**	**0.880**	**0.945**
TAU		0.805	0.067	1.339	0.18	0.893	0.859	0.922
PsychoDynamic		0.769	0.079	0.563	0.57	0.884	0.842	0.920
CTBE*		**0.320**	**0.127**	**−3.699**	**<0.001**	**0.748**	**0.658**	**0.829**
Specified other*		**0.827**	**0.062**	**2.034**	**0.042**	**0.898**	**0.868**	**0.924**
CBT		0.691	0.121	−0.358	0.72	0.864	0.793	0.920
Treatment category × quarter*								
Quarter 1	DBT	0.431	0.064	−0.702	0.48	0.785	0.743	0.826
	DBTreduced	0.423	0.094	−0.562	0.57	0.783	0.719	0.841
	ST*	**0.872**	**0.086**	**4.989**	**<0.001**	**0.908**	**0.868**	**0.941**
	TFP	0.309	0.134	−1.412	0.16	0.744	0.649	0.830
	MBT*	**1.066**	**0.121**	**5.453**	**<0.001**	**0.945**	**0.899**	**0.975**
	TAU	0.448	0.073	−0.426	0.67	0.791	0.742	0.836
	PsychoDynamic	0.554	0.098	0.891	0.37	0.824	0.762	0.879
	CTBE*	**−0.221**	**0.161**	**−4.832**	**<0.001**	**0.551**	**0.443**	**0.667**
	Specified Other*	**0.607**	**0.070**	**2.142**	**0.033**	**0.840**	**0.798**	**0.878**
	CBT	0.263	0.128	−1.854	0.06	0.728	0.637	0.812
Quarter 2	DBT	0.629	0.069	−0.984	0.33	0.847	0.805	0.883
	DBTreduced	0.566	0.119	−1.108	0.27	0.828	0.752	0.892
	ST*	**0.957**	**0.093**	**2.917**	**0.004**	**0.926**	**0.886**	**0.956**
	TFP	0.675	0.154	−0.152	0.88	0.860	0.766	0.930
	MBT*	**0.959**	**0.112**	**2.615**	**0.009**	**0.926**	**0.877**	**0.961**
	TAU	0.811	0.093	1.390	0.17	0.895	0.847	0.933
	PsychoDynamic	0.701	0.106	0.050	0.96	0.867	0.806	0.916
	CTBE*	**0.202**	**0.182**	**−2.970**	**0.003**	**0.706**	**0.575**	**0.826**
	Specified Other	0.798	0.078	1.445	0.15	0.891	0.851	0.925
	CBT	0.662	0.209	−0.189	0.85	0.856	0.724	0.946
Quarter 3	DBT	0.700	0.081	−1.753	0.08	0.866	0.820	0.906
	DBTreduced*	**0.514**	**0.166**	**−2.088**	**0.037**	**0.812**	**0.701**	**0.901**
	ST*	**1.233**	**0.111**	**3.610**	**<0.001**	**0.968**	**0.937**	**0.986**
	TFP	0.905	0.173	0.392	0.70	0.915	0.828	0.969
	MBT	0.809	0.107	−0.331	0.74	0.894	0.838	0.937
	TAU	0.946	0.120	0.922	0.36	0.924	0.869	0.962
	PsychoDynamic	0.827	0.131	−0.127	0.90	0.898	0.829	0.948
	CTBE	0.703	0.216	−0.704	0.48	0.867	0.733	0.954
	Specified Other	0.890	0.088	0.584	0.56	0.912	0.872	0.945
	CBT	0.901	0.263	0.250	0.80	0.915	0.770	0.984
Quarter 4	DBT	0.919	0.094	0.163	0.87	0.919	0.876	0.951
	DBTreduced	0.708	0.192	−1.075	0.28	0.869	0.752	0.948
	ST*	**1.134**	**0.107**	**2.170**	**0.030**	**0.955**	**0.920**	**0.978**
	TFP	0.930	0.170	0.161	0.87	0.921	0.838	0.971
	MBT	0.798	0.108	−1.028	0.30	0.891	0.834	0.936
	TAU	1.017	0.131	0.893	0.37	0.937	0.882	0.972
	PsychoDynamic	0.995	0.143	0.682	0.50	0.933	0.870	0.972
	CTBE	0.596	0.213	−1.574	0.12	0.837	0.697	0.936
	Specified Other	1.012	0.099	1.157	0.25	0.936	0.896	0.965
	CBT	0.939	0.268	0.142	0.89	0.922	0.780	0.987

Significant effects in **bold**. * *p* < 0.05.

**Fig. 3.**
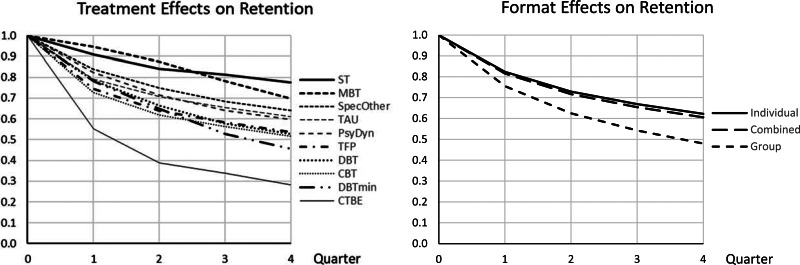
Retention curves for 4 quarters for the complete data set. (*a*) (left). Cumulative treatment retention over 4 quarters depicted with survival curves for the 10 treatment models. Over 1 year CTBE had considerable less treatment retention, while ST and MBT had considerable more. (*b*) (right). Cumulative treatment retention over 4 quarters depicted with survival curves for the 3 treatment formats. Over 1 year group formats had considerable less treatment retention than the other two.

Pure or predominantly group treatments had significantly less than average treatment retention. Studies reporting not enough details to infer dropout per quarter were associated with less retention. The significant main effect of treatment model and the treatment model by quarter interaction were related to the following specific effects. ST had a significantly higher treatment retention in all quarters. MBT had a significantly higher treatment retention than average in Quarter 1 and 2, Specified Others in Quarter 1, effects that disappeared in later quarters. CTBE had lower treatment retention in Quarter 1 and 2, CBT in Quarter 1, and reduced DBT in Quarter 3. Figure 3 shows the (cumulative) survival curves per treatment model (3a, left panel) and format (3b, right panel). After one year, the (unweighted) average retention was 57%, with CTBE showing considerably lower (28%) and ST considerably higher treatment retention (78%).

#### Funnel plot

**Fig. 4.**
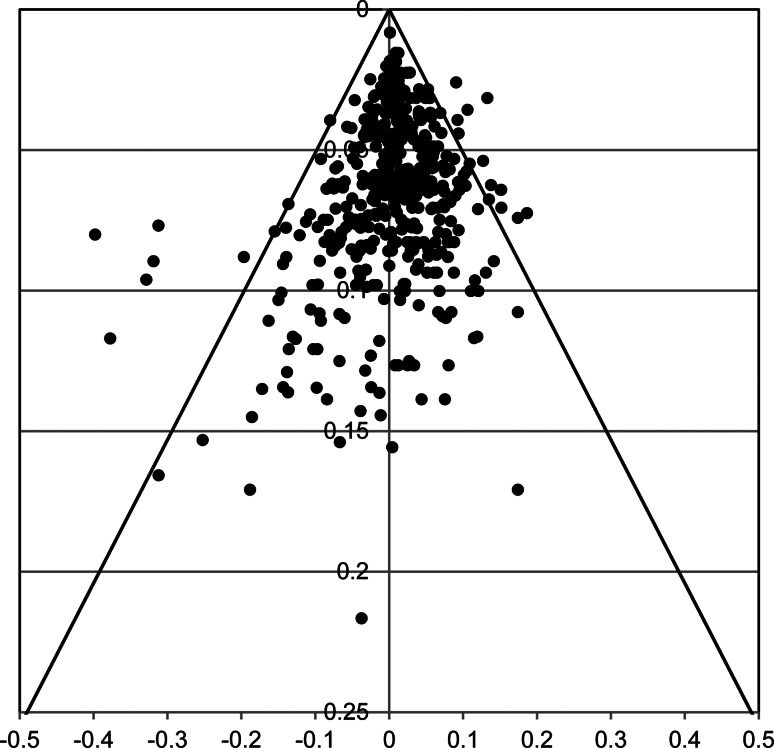
Funnel plot of 463 residuals of the final GLMM survival analysis (*x*-axis = residual; *y*-axis = study precision per quarter). Residuals were the differences between observed and estimated survival proportions. To the left residuals related to more actual dropouts in a quarter than predicted by the model, to the right residuals related to less actual dropouts than predicted by the model. There were 23 (4.96%) residuals outside the 95% CI.

Figure 4 presents the funnel plot over all treatments arms and quarters (Appendix G presents funnel plots per treatment arm). Note that each value represents a residual of a specific quarter of a specific arm of a study. There were 23 residuals lying outside the 95% CI, which is 4.96% of the 463 residuals – thus less than the 5% that can be expected given the 95% CI. Nine of the outliers (to the left) were related to more actual dropouts than predicted by the GLMM survival model: three DBT (Barnicot & Crawford, 2019, Quarter 4; Fitzpatrick, Bailey, & Rizvi, 2020, Quarter 2; Sinnaeve, van den Bosch, Hakkaart-van Roijen, & Vansteelandt, 2018, Quarter 1); two TAU (Soler et al., 2009, Quarter 1; Verheul et al., 2003, Quarter 1); one psychodynamic (Löffler-Stastka, Ponocny-Seliger, Meißel, & Springer-Kremser, 2006, Quarter 1); one CTBE (Doering et al., 2010, Quarter 1); one specified other (Chanen et al., 2022, Quarter 1), and one CBT (Morey, Lowmaster, & Hopwood, 2010, Quarter 1). Seven of 9 were from Quarter 1. Fourteen outliers (to the right) were related to less actual dropouts than predicted, all from relatively more precise observations: four DBT (Fitzpatrick, 2020, Quarter 1; Sinnaeve, 2018, Quarter 3; Verheul et al., 2003, Quarter 4; Walton, Bendit, Baker, Carter, & Lewin, 2020, Quarter 1); one MBT (Barnicot & Crawford, 2019, Quarter 3); five TAU (Bos, van Wel, Appelo, & Verbraak, 2010, Quarter 1; Carter, Willcox, Lewin, Conrad, & Bendit, 2010, Quarter 2; Kleindienst et al., 2011; Quarter 1; Majdara, Rahimian-Boogar, Talepasand, & Gregory, 2021, Quarter 1; Priebe et al., 2012, Quarter 1), one CTBE (Linehan et al., 2006, Quarter 1); two Specified Other (Chanen et al., 2022, Quarter 1 & 4); and one CBT (Cottraux et al., 2009, Quarter 1). Again, most outliers were from Quarter 1 (9/14). Two outpatient DBT study-arms had outliers of different signs (at different quarters; Fitzpatrick 2020; Sinnaeve 2018), indicating rather the timing of dropout than the cumulative dropout was diverting from the model. Given the heterogeneous character of TAU, it is understandable that relatively many outliers (7/23) came from the TAU category. Taken together, the number of outliers is in the expected range, but whereas outliers indicating underestimation of treatment retention were at the higher precision level, outliers indicating overestimation of retention were at a more medium precision level. This is in line with the results of Egger's test. Note that no study had quarters with residuals systematically outside the 95% CI.

#### Sensitivity analysis: DBT studies with deviating pushout rules excluded

**Fig. 5.**
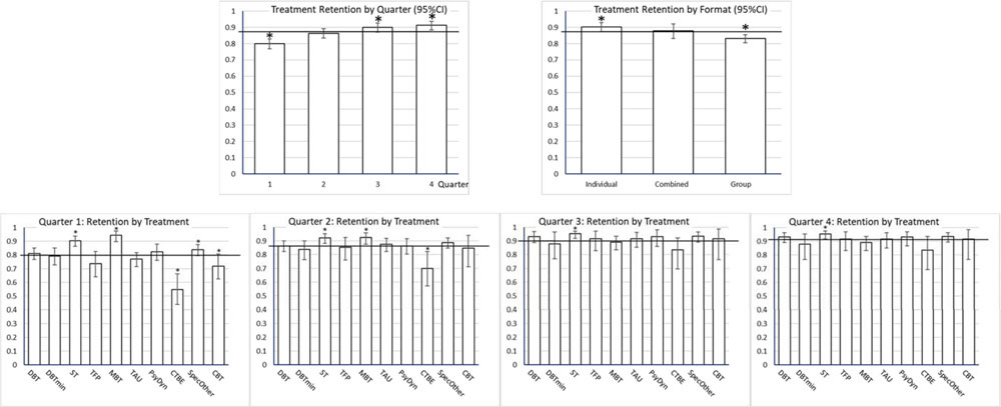
Treatment retention proportion per quarter (with 95% CI) as estimated in the reduced dataset, without DBT-arms with deviating pushout rules. The horizontal line is the average treatment retention, to which the estimated effects are compared (deviation contrasts). Significant effects (*p* < 0.05) indicated by *. Upper left panel: treatment retention by quarter, showing increasing retention with time. Upper right panel: treatment retention by treatment format, illustrating significantly less retention in group and more in individual treatment. Lower panels: treatment retention by treatment types and quarter. In all quarters, ST had significantly higher treatment retention than average. In quarters 1 and 2 MBT had significantly higher retention, CTBE significantly less, than average. In Quarter 1, specified others had significantly more and CBT less retention than average.

Two British DBT studies used a pushout rule deviating from the rule as formulated in the DBT protocol: participants were pushed out when they missed any consecutive series of 4 sessions, for instance 2 skills group and 2 individual coaching sessions within 2 weeks; whereas the original guideline is 4 consecutive sessions of either group or individual (Barnicot & Gaglia, personal communication, 24 September 2016). The more stringent rule used in the two studies (Gaglia et al., 2013; Priebe et al., 2012) seems related to relatively high dropout (Appendix D). We therefore repeated the analyses with the DBT arms of these studies excluded. The initial model, before backward deletion, was highly similar to the one based on the complete study set, except that treatment format was significant (Table 4). The random effect of study was significant, z = 4.889, *p* < 0.001 (*I*^2^ = 100*.146/1.146 = 12.7%). Backward deletion resulted in the same set of predictors (quarter, treatment, treatment format and dropout imputation) as in the primary analysis (Table 4). For the final model only the treatment by quarter interaction was added, Table 4. Egger's test was significant, explaining 3.9% of the variance, Table 4. The random effect of study was significant, z = 5.286, *p* < 0.001 (*I*^2^ = 100*.120/1.120 = 10.7%). Figure 5 shows the fixed effects of the final model, Table 5 the statistics of the deviation contrasts.

**Table 4.** *F*-tests of predictors of treatment retention of the fixed part of the initial, intermediate, and final models, without DBT-arms of Priebe and Gaglia studies

Model	*F*	df1	df2	*p*
Initial model				
Corrected model	6.753	33	179	<0.001
Year of publication	1.753	1	70	0.19
Methodological quality of study's arm	0.081	1	120	0.78
Trial type	0.454	2	75	0.64
Dropout type distinguished	0.495	1	67	0.48
TAU added to treatment	0.602	1	632	0.44
Dropout per quarter to be imputed*	**5.705**	**1**	**137**	**0.018**
Setting (inpatient, day treatment, outpatient)	1.646	2	273	0.20
**Treatment format (Individual, Group, Combined)**	**3.344**	**2**	**1278**	**0.036**
Medicine prescribed by study	0.985	1	80	0.32
Substance use exclusion	0.535	4	70	0.71
Country group (Europe, USA, Aus/Can/NZ, emerging)	0.266	3	68	0.85
Mean age of study's arm	0.077	1	539	0.78
Proportion males of study's arm	0.067	1	375	0.80
Treatment category*	**4.975**	**9**	**987**	**<0.001**
Quarter*	**47.817**	**3**	**22 986**	**<0.001**
Intermediate model (Main Effects after backwards deletion)				
Corrected model	14.614	15	1335	<0.001
Dropout per quarter to be imputed*	**5.528**	**1**	**136**	**0.020**
Treatment format (Individual, Group, Combined)*	**4.718**	**2**	**944**	**0.009**
Treatment category*	**6.194**	**9**	**1754**	**<0.001**
Quarter*	**49.073**	**3**	**23 004**	**<0.001**
Final main and interaction effects model				
Corrected model	7.007	42	4441	<0.001
Dropout per quarter to be imputed*	**5.648**	**1**	**131**	**0.019**
Treatment format (Individual, Group, Combined)*	**4.947**	**2**	**959**	**0.007**
Treatment category*	**4.811**	**9**	**2248**	**<0.001**
Quarter*	**36.769**	**3**	**22 977**	**<0.001**
Treatment category × quarter*	**2.757**	**27**	**22 977**	**<0.001**
Egger's test (1/√(*N*)*	**6.453**	**1**	**159**	**0.012**

**Table 5.** Nominal Predictors: Retention chances and follow-up contrasts of the final model [reduced study set (without DBT arms from Priebe 2012 and Gaglia et al., 2013)]

		Transformed scale				Estimated survival chance per quarter		
Predictor		Pooled mean	Rubin's s.e.	Deviation contrast		Pooled mean	95% CI	
				*t*	*P*		Upper	Lower
Dropout per quarter imputed*								
No*		**0.808**	**0.050**	**2.266**	**0.025**	**0.894**	**0.869**	**0.916**
Yes*		**0.645**	**0.066**	**−2.266**	**0.025**	**0.851**	**0.813**	**0.886**
Treatment format*								
Individual*		**0.850**	**0.056**	**2.612**	**0.009**	**0.904**	**0.877**	**0.927**
Combined individual-group		0.750	0.054	0.666	0.51	0.880	0.851	0.905
Group*		**0.579**	**0.078**	**−2.988**	**0.003**	**0.832**	**0.784**	**0.875**
Quarter*								
Quarter 1*		**0.473**	**0.049**	**−9.134**	**<0.001**	**0.799**	**0.767**	**0.829**
Quarter 2		0.692	0.055	−1.033	0.30	0.864	0.833	0.892
Quarter 3*		**0.841**	**0.062**	**3.112**	**0.002**	**0.901**	**0.872**	**0.927**
Quarter 4*		**0.899**	**0.064**	**4.473**	**<0.001**	**0.914**	**0.885**	**0.938**
Treatment category*								
DBT		0.753	0.064	0.459	0.65	0.880	0.846	0.910
DBTreduced		0.591	0.097	−1.454	0.15	0.836	0.776	0.887
ST*		**1.033**	**0.073**	**4.801**	**<0.001**	**0.940**	**0.913**	**0.961**
TFP		0.689	0.105	−0.416	0.68	0.864	0.802	0.913
MBT*		**0.910**	**0.082**	**2.667**	**0.008**	**0.917**	**0.880**	**0.946**
TAU		0.726	0.071	0.005	1.00	0.873	0.834	0.907
PsychoDynamic		0.765	0.081	0.548	0.58	0.883	0.840	0.919
CTBE*		**0.311**	**0.128**	**−3.176**	**<0.001**	**0.745**	**0.654**	**0.827**
Specified other		0.818	0.063	1.898	0.06	0.896	0.865	0.923
CBT		0.664	0.123	−0.568	0.57	0.857	0.783	0.916
Treatment category × quarter*								
Quarter 1	DBT	0.510	0.068	0.572	0.57	0.811	0.768	0.851
	DBTreduced	0.453	0.096	−0.209	0.83	0.793	0.728	0.850
	ST*	**0.858**	**0.087**	**4.811**	**<0.001**	**0.905**	**0.863**	**0.939**
	TFP	0.294	0.135	−1.517	0.13	0.738	0.643	0.826
	MBT*	**1.067**	**0.121**	**5.463**	**<0.001**	**0.945**	**0.899**	**0.975**
	TAU	0.385	0.076	−1.331	0.18	0.770	0.718	0.818
	PsychoDynamic	0.554	0.010	0.905	0.37	0.824	0.761	0.879
	CTBE*	**−0.232**	**0.162**	**−4.865**	**<0.001**	**0.548**	**0.439**	**0.663**
	Specified other*	**0.598**	**0.071**	**2.006**	**0.045**	**0.838**	**0.795**	**0.876**
	CBT*	**0.243**	**0.130**	**−1.986**	**0.048**	**0.721**	**0.628**	**0.807**
Quarter 2	DBT	0.696	0.073	0.059	0.95	0.865	0.824	0.901
	DBTreduced	0.601	0.121	−0.771	0.44	0.839	0.763	0.901
	ST*	**0.942**	**0.093**	**2.788**	**0.005**	**0.923**	**0.882**	**0.954**
	TFP	0.659	0.155	−0.238	0.81	0.855	0.760	0.927
	MBT*	**0.964**	**0.113**	**2.699**	**0.007**	**0.927**	**0.878**	**0.962**
	TAU	0.741	0.097	0.571	0.57	0.877	0.824	0.921
	PsychoDynamic	0.696	0.107	0.046	0.96	0.866	0.803	0.916
	CTBE*	**0.194**	**0.182**	**−2.994**	**0.003**	**0.703**	**0.572**	**0.824**
	Specified other	0.789	0.079	1.367	0.17	0.889	0.848	0.923
	CBT	0.637	0.212	−0.304	0.76	0.849	0.713	0.943
Quarter 3	DBT	0.809	0.088	−0.368	0.71	0.894	0.849	0.930
	DBTreduced	0.557	0.168	−1.789	0.07	0.825	0.715	0.911
	ST*	**1.216**	**0.112**	**3.459**	**<0.001**	**0.966**	**0.933**	**0.985**
	TFP	0.888	0.174	0.302	0.76	0.912	0.823	0.967
	MBT	0.811	0.108	−0.291	0.77	0.895	0.838	0.938
	TAU	0.861	0.126	0.172	0.86	0.906	0.842	0.951
	PsychoDynamic	0.822	0.133	−0.155	0.88	0.897	0.827	0.948
	CTBE	0.695	0.217	−0.731	0.46	0.865	0.730	0.953
	Specified other	0.880	0.088	0.473	0.64	0.910	0.868	0.943
	CBT	0.869	0.267	0.121	0.90	0.908	0.757	0.982
Quarter 4	DBT	0.998	0.101	1.034	0.30	0.934	0.892	0.963
	DBTreduced	0.752	0.193	−0.796	0.43	0.880	0.766	0.955
	ST*	**1.119**	**0.108**	**2.067**	**0.039**	**0.953**	**0.916**	**0.977**
	TFP	0.914	0.170	0.098	0.92	0.918	0.832	0.969
	MBT	0.800	0.109	−0.947	0.34	0.892	0.834	0.937
	TAU	0.919	0.137	0.152	0.88	0.918	0.853	0.962
	PsychoDynamic	0.988	0.144	0.672	0.50	0.932	0.868	0.972
	CTBE	0.588	0.213	−1.583	0.11	0.835	0.694	0.935
	Specified other	1.004	0.100	1.128	0.26	0.935	0.894	0.964
	CBT	0.908	0.272	0.035	0.97	0.916	0.766	0.985

Significant effects in bold. * *p* < 0.05.

The results of the deviation contrasts can be described as follows.

**Fig. 6.**
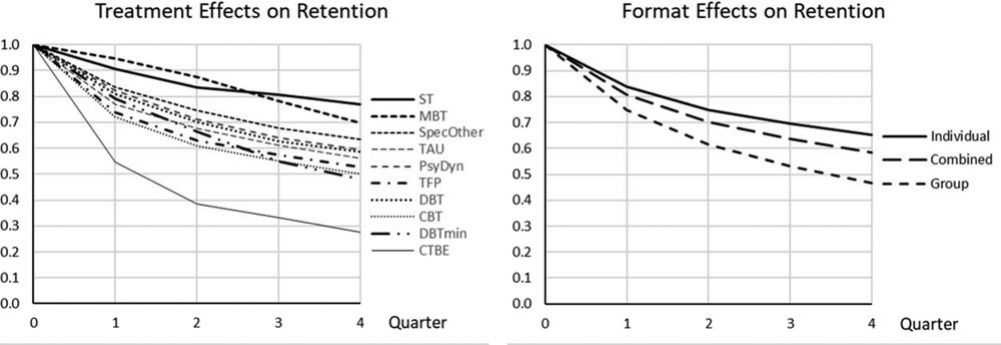
Retention curves for 4 quarters for the reduced data set (sensitivity analysis). (*a*) (left). Cumulative treatment retention over 4 quarters depicted with survival curves for the 10 treatment models, estimated from the reduced data set, without DBT-arms with deviant pushout rules. Over 1 year CTBE had considerable less treatment retention, while ST and MBT had considerable more. (*b*) (right). Cumulative treatment retention over 4 quarters depicted with survival curves for the 3 treatment formats, estimated from the reduced data set, without DBT-arms with deviant pushout rules. Over 1 year group formats had considerable less treatment retention, while individual had considerably more treatment retention than average. The combined format was in between.

1. *Quarter.* As in the primary analysis, the first quarter had the lowest retention, with later quarters showing increasing levels of treatment retention.
2. *Dropout imputation per quarter* was significantly related to less retention.
3. *Treatment models.* As in the primary analysis, ST and MBT showed generally higher and CTBE less treatment retention than average. As to the treatment by quarter interaction, the results were mostly similar compared to the primary analysis, with the exception that reduced DBT did no longer show lower treatment retention in quarter 3. Figure 6a shows the (cumulative) survival curves per treatment. After one year, the (unweighted) average retention was about 57%, with CTBE showing considerably lower (28%), MBT and ST higher treatment retention (70%, 77%).
4. *Treatment format.* Individual treatment had significantly higher and group significantly lower than average treatment retention, combined individual-group format in between the other two. The relationship was approximately linear: the stronger the group component, the lower treatment retention was. Figure 6b shows the survival curves for the three formats over 1 year. At 1 year, the retention estimate was 66.5% for individual, 60% for combined, and 48% for group format.

**Fig. 7.**
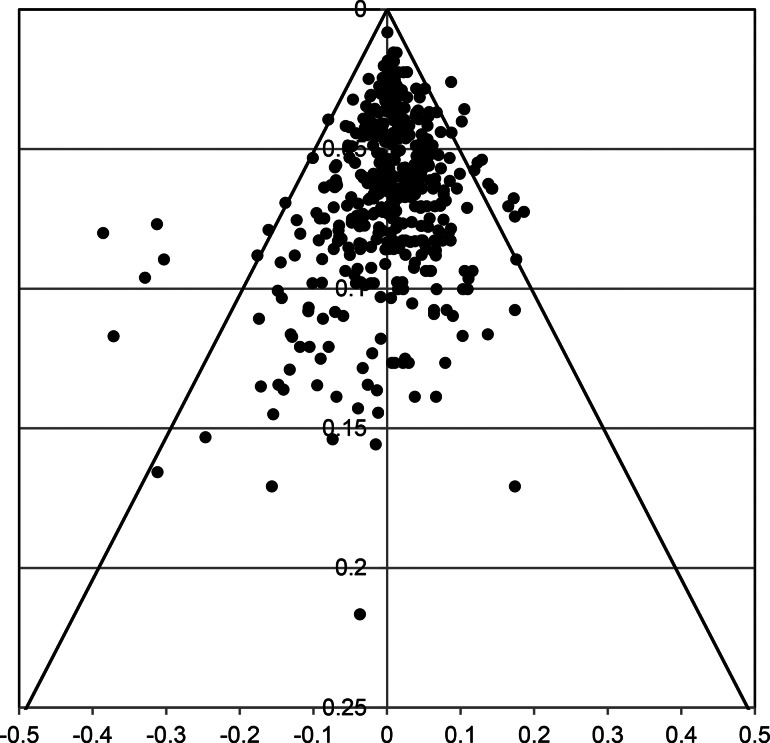
Funnel plot of 455 residuals of the final GLMM survival analysis (*x*-axis = residual; *y*-axis = study precision per quarter) of the reduced data set. Residuals were the differences between observed and estimated survival proportions. To the left residuals related to more actual dropouts in a quarter than predicted by the model, to the right residuals related to less actual dropouts than predicted by the model. There were 24 (5.3%) residuals outside the 95% CI.

Figure 7 depicts the funnel plot of the residuals of the final analysis of the reduced study set (see Appendix H for funnel plots per treatment category). There were 24 outliers out of a total of 455 residuals (5.3%). Most outliers were the same as in the full data analysis, however one disappeared (Priebe 2012, TAU, Q1) and two additional positive residuals emerged (Barnicot & Crawford, 2019, MBT, Q4; Sachdeva, Goldman, Mustata, Deranja, & Gregory, 2013, TAU, Q1). Most were from quarter 1 (16/24).

## Discussion

Premature treatment discontinuation is a well-known phenomenon in the treatment of BPD. It has not only plagued health care of BPD-patients for years, but has also motivated the development of specialized approaches, like the ‘big-4’, that were designed to (among other things) reduce treatment dropout. We used a meta analytic approach to study treatment retention in psychological therapies for BPD, and tested various factors that might be associated with treatment retention. We found evidence for superior treatment retention in MBT and ST. CTBE showed very poor treatment retention, mainly in the first two quarters of treatment. Specified others showed somewhat higher retention than average in Quarter 1, CBT lower retention in Quarter 1 and reduced DBT in Quarter 3 (both in one of the two analyses). All other treatment categories did not differ significantly from the average. We did not find any evidence that on average over the last 32 years treatment retention improved, nor did we find evidence that gender or patients' age had any influence. No effect of treatment setting was detected, with no significant differences between inpatient, outpatient and day-treatment settings. However, there was evidence for an effect of treatment format, with group therapy having higher dropout than other formats. Interestingly, when the two DBT arms that used a more stringent pushout rule than the original DBT protocol were excluded from the analysis, individual therapy had the lowest and group therapy the highest dropout, with mixed individual-group approaches having average retention. Study design (RCT, open trial, nonrandomized controlled), design quality, dropout type (treatment dropout *v.* no distinction made between treatment and study dropout), prescribed medication policy, treatment offered in addition to TAU, country group, and type of exclusion of substance use, had no significant effects on dropout rates. However, insufficient details about timing of treatment dropouts (necessitating MI to estimate dropout per quarter) had a significant effect in that these studies were associated with less treatment retention. By including this study characteristic as a covariate we controlled for this effect. Last, but not least, we found that it is especially the first quarter of treatment during which dropout manifests itself.

Egger's test and Funnel plots indicated that less precise studies, such as studies with a small sample size, were associated with *less* treatment retention. Note that this is opposite to what might be expected in case of (publication) bias, where less precision would be expected to be associated with overly optimistic findings, i.e. higher treatment retention. Moreover, though significant, the effect was small (<4% explained variance). The number of residuals exceeding a magnitude that could be expected on the basis of a 95% CI was around the to be expected 5%. Most of the extreme residuals came from the first Quarter and from treatment categories that were heterogeneous, such as TAU, CTBE, and specified others. With seven exceptions of DBT and one of MBT, none of the ‘Big-four’ residuals was excessive. Thus, treatment dropout could be estimated fairly precisely for these four specialized psychotherapies.

Excluding the DBT-arms of the Priebe and Gaglia studies as was done in the sensitivity analysis might yield more trustworthy results than an analysis including these arms. Interestingly, an effect of individual therapy format appeared in this analysis, indicating that the stronger the individual component of the treatment, the higher the retention rate is. A recent RCT compared predominantly group to combined individual-group treatment and also found more dropout from the predominantly group format, supporting a causal interpretation of format (Arntz et al., 2022).

Our study differs from previous studies documenting dropout from psychological treatment for BPD (e.g. Barnicot et al., 2011; Iliakis, Ilagan, & Choi-Kain, 2021; Stoffers-Winterling et al., 2012; Storebø et al., 2020). For instance, in contrast to the Cochrane meta-analysis by Stoffers-Winterling et al. (2012) and Storebø et al. (2020), we included all kinds of designs, investigated the development of retention over time (and not accumulated over time), included multiple predictors (i.e. meta-regression), and based the analysis on individual cases (thus, in a sense our study was a ‘mega-analysis’, although for some predictors we did not have individual values). In contrast to the approach chosen in the Cochrane analysis, our approach allowed comparison between treatment models and formats. Although the meta-analysis by Barnicot et al. (2011) distinguished between treatments with a duration shorter than one year *v.* longer treatments, the survival analysis approach we used was more fine-graded in its modeling of the development of dropout over time. Moreover, we found that in addition to time (quarter), treatment format and treatment model were important predictors. Our findings suggest that the substantial between-study heterogeneity found by Barnicot et al. (2011) and Iliakis et al. (2021) can be explained for an important part by these variables. Note that the pooled completion rates found by Barnicot et al. (2011) are similar to those we found.

### Clinical implications

The results suggest some important implications for clinical practice. First, as most dropout takes place in the first quarter, it is pivotal to give attention at the start of treatment to factors that influence treatment engagement. Although this has been acknowledged in some specialized models (e.g. the ‘contract phase’ in TFP), the present results do not support that all attempts are successful. More research is needed to understand why patients tend to dropout so early in treatment and how treatment can be made more acceptable, especially in the early phase. Second, pure individual treatment has superior retention, whereas the larger the group part in treatment is, the lower treatment retention is. Factors like practical (e.g. agenda) problems, but also group-dynamics might play a role here. Although mixed individual-group models seem to do better, it is the pure individual treatment that has the highest treatment retention. The tendency to provide more therapies in group formats in an attempt to reduce delivery costs might thus result in higher personal and societal costs associated with dropout. More research is needed to understand what underlies dropout from group treatment, what can be done to prevent this, and, perhaps, how patients can be better matched to group, combined, or individual treatment. Better understanding of factors that are involved in dropout from groups is even more important when patients will learn about the higher dropout risk from group treatment, as this might increase resistance against groups. As not all patients drop out from groups, and the treatments with the highest retention, MBT and ST, involve group components (for ST the recent models) there might be more factors involved than the group modality as such. For example, the degree of structure and hence safety in the group probably plays an important role in preventing dropout. On the other hand, the format effect should not be underestimated: even for ST the difference between individual (87% retention over 1 year) and group (73% retention) is large. Pending research that will help to better personalize the matching of treatment format to patients, a clinical recommendation might be to take resistance to group treatment seriously and consider individual treatment for those that indicate to be too distrustful, inhibited, or easily provoked (in anger or aggression) to participate in a group. In other words, it is suggested to explore with the patient whether a group format is a good match with the patient instead of mechanistically putting everyone in a group treatment for reasons of efficiency.

The finding that more than 30 years of research has not led to a general improvement of treatment retention is disappointing. However, during this period new specialized treatment models were developed and tested, and the present evidence suggests that some, but not all of them, might actually prevent premature treatment discontinuation. The results therefore do not support claims that all (specialized) approaches are equal (e.g. Paris, 2010). If dropout systematically differs between treatment approaches, there must be specific factors that account for this. In the absence of equivalence trials (or equivalence meta-analysis), claims that treatments are equivalent, typically based on nonsignificant differences between treatments, were premature anyway. The present findings cast serious doubt on such claims, at least with regard to treatment retention.

The finding that CTBE has such a high dropout rate is remarkable, especially because CTBE has been framed as a superior variant of TAU. One interpretation is that patients agree to participate in a trial involving CTBE in the hope to get the experimental and not the control treatment, and drop out if they do not get the preferred treatment. However, one would then expect a similar finding with TAU, which was not the case. Possibly it is the strong focus on addressing difficult issues of the patient by CTBE therapists using traditional, confrontational psychotherapeutic strategies that makes the treatment difficult to tolerate.

More traditional psychodynamic treatment than for instance TFP and MBT did not show inferior treatment retention. This is surprising, given early findings that psychodynamic psychotherapy with BPD-patients was associated with high dropout (Gunderson et al., 1989; Skodol et al., 1983; Waldinger & Gunderson, 1984). These early studies however used a wider definition of BPD, than that of the DSM-III and later editions, and thus might have included more difficult patients. Moreover, the presently included studies of psychodynamic treatments might have investigated (successful) adaptations of psychodynamic psychotherapy, e.g. involving a more supportive and less neutral stance of therapists, that differ from the more traditional versions that were investigated in early studies.

The pooled treatment retention in DBT was strongly influenced by two studies using a stricter pushout rule than the DBT protocol prescribes. Excluding the two studies from the analysis led to an increase in estimated treatment retention in DBT (estimated survival chance over 1 year increased from 53% to 59%). Taken together, the present data indicate that the original DBT protocol, and not more stringent rules should be followed when it comes to prevent treatment dropout.

The finding that ST has low dropout rates is in line with previous observations, both in BPD and in other PDs. For instance, RCTs for non-borderline PDs have also found superior treatment retention in ST compared to other treatments (Bamelis, Evers, Spinhoven, & Arntz, 2014; Bernstein et al., 2021). What might explain the high treatment retention in ST? First, patients tend to appreciate the therapeutic relationship in ST higher than in comparison conditions, and higher appreciation is associated with treatment retention (Bamelis et al., 2014; Spinhoven, Giesen-Bloo, van Dyck, Kooiman, & Arntz, 2007). Second, qualitative research into patients' perspectives suggests a number of elements in ST that are particularly appreciated by patients: (i) the schema mode model, helping patients to better understand and control their problems; (ii) the therapeutic relationship: which is experienced as more personal, directive and caring than in other models; and (iii) specific ST techniques, notably experiential techniques, such as imagery rescripting, which are reported as particularly helpful (de Klerk, Abma, Bamelis, & Arntz, 2017). Third, in contrast to what was found with other treatments (Katsakou et al., 2012), patients did not report a too narrow focus of ST (de Klerk et al., 2017). Thus, it seems that the ST model meets the needs of patients quite well. ST for BPD has now been studied in 12 studies, in 7 countries, by different research groups. Clearly, this still limited database calls for further studies that will help to clarify whether ST indeed is characterized by a high treatment retention.

ST was not the only treatment model showing superior treatment retention: in the first two quarters of treatment, this was also shown by MBT. However, the data indicate that the initial high retention chance in MBT is not maintained in later phases of treatment, whereas ST continued to show the highest retention chance per quarter. Nevertheless, cumulative treatment retention over 1 year was also relatively high in MBT (70% *v.* 78% in ST)

### Limitations

A number of limitations of the present meta-analysis should be considered. First, a meta-analysis is not an RCT – hence differences in populations and sites might influence findings. This might especially be a problem when not all treatment approaches (including treatment models, formats and settings) are directly compared in RCT's. On the other hand, we did not find evidence that the study design (RCT *v.* non-RCT *v.* open trial) influenced results. Moreover, not enough direct comparisons between treatments and formats are available, and it would be a virtually impossible task to compare 16 treatment models each provided in 3 formats, each delivered in 3 settings, thus 144 arms to each other in multiple RCTs (i.e. 10 296 comparisons). The current analysis provides a statistical summary, not a proof, of what studies into psychological treatment of BPD found with respect to treatment retention. With all the problems that are inherent to a meta-analysis, the current study indicates what is associated with treatment retention, and what not, and thus informs clinical practice and researchers.

Second, estimation of (parts of) the development of dropout over time was necessary in many studies. Although we used MI as an appropriate statistical strategy to deal with this, it is always better to base the analysis on the original detailed survival lengths. Future trials should follow guidelines such as the CONSORT statement (Schulz, Altman, & Moher, 2010) and report treatment retention in sufficient detail.

Third, we could not always distinguish between treatment and study dropout. Although we did not find a significant effect of not distinguishing between study and treatment dropout, future studies should follow guidelines of reporting by distinguishing between dropout types (e.g. CONSORT-guidelines, Schulz et al., 2010).

Fourth, only two studies had a CTBE arm, limiting the generalizability of the CTBE findings, though the two studies were from different continents and reported remarkably similar findings. Nevertheless, the high dropout rates from CTBE are alarming, and questions both the ‘expert’ level of the therapists and the suitability of CTBE as comparison condition in RCTs.

Fifth, some treatment models, such as DBT, have rules about pushouts, while others have not. Such rules are defined by the treatment protocol however, and are based on the model underlying the protocol. Thus, we considered the effects of such rules on treatment retention as inherent to these models. Future research should test whether changes in such rules lead to changes in treatment retention, or not.

Sixth, the influence of covariates such as gender and age could only be assessed on a aggregated level. This not only reduced the power of our statistical tests of these variables, but also has the risk that effects are overseen as within-study relationships might not be detected when means of studies are analyzed (Simpson's paradox). Analysis of individual data would solve this problem, but requires data sharing between researchers. Another issue that might limit the validity of the results on the influence of gender is the average 15% of male participants in the data. This is very low compared to prevalence estimates from the general population, that generally show equal prevalence in men and women (Torgersen, 2012). Samples from mental health centers generally show a dominance of female patients (about 75%, American Psychiatric Association, 2013). The difference in gender proportion might be related to the use of structured interviews by lay interviewers in epidemiological research (*v.* semi-structured interviews by clinicians in clinical samples), gender differences in help seeking behavior, and higher numbers of men with BPD in addiction treatment centers and forensic institutes. But even compared to the approximately 25% prevalence of men in clinical samples the 15% in the present data is low, possibly related to the habit to recruit female patients only by some researchers (e.g. Linehan et al., 2015). Thus, the finding that mean age and mean proportion of male patients did not relate to treatment retention should be interpreted with caution.

Seventh, we collapsed treatment models with sample size less than 100 into one category. The resulting specialized others category is quite heterogeneous, and conclusions about specific treatment models within this category cannot be drawn. The relatively high retention in Quarter 1 suggests that there might be promising treatments in this category.

Eighth, keeping patients in treatment that will not profit from it, is not cost-effective: the resources can better be allocated to those that will profit. Thus, high treatment retention as such is not necessarily good. On the other hand, the (cost-)effectiveness of treatments is limited by premature dropouts, as dropout limits the potential overall (cost)effectiveness. High treatment retention does not necessarily imply high effectiveness, however in a recent meta-analysis on effectiveness using a similar multilevel approach as the current study we found evidence for relatively high effect sizes of ST and MBT (and lower effectiveness of TAU and CTBE; Rameckers et al., 2021).

Ninth, the team that performed the present meta-analysis was led by the first author, an ST expert, which raises the question whether allegiance effects influenced the findings. To prevent such effects, study selection was done by different combinations of 3 from 4 experts and although the first author co-selected studies, others were the majority, and none of them had an affiliation with ST. The first author did not assess quality nor coded dropout data and other study characteristics, and none of the coders had an ST-affiliation. The analyses were collaboratively done by the last author, a statistician not affiliated with any treatment, and the first author. Lastly, the data are given in the appendices so that other researchers can check them and conduct independent analyses.

## Conclusions

Although the current findings are not a definitive proof that treatments models and formats differ in treatment retention, they are provocative and help to stimulate further research into improving treatment retention for BPD patients. Contradicting the popular ‘Dodo bird verdict’ that all treatments are equal, the findings suggest that they are not when it comes to treatment retention. More specifically, CTBE does not seem to be a good idea, whereas ST and MBT seem to do a better job than other treatments. Individual treatment seems to protect against dropout, whereas group treatment in particular might be a risk factor for premature treatment ending. Some factors thought to be predictive of dropout were not supported, and it might not be recommended to use them for treatment selection.

## Footnotes

† The notes appear after the main text.

## Notes

1 Thanks are due to Dr. Stephan Doering for raising this hypothesis during a discussion at the ESSPD conference in Vienna, 8–10 September 2016.

2 Thanks are due to Dr. Martin Bohus for raising this hypothesis during a discussion at the ESSPD conference in Vienna, 8–10 September 2016.

3 SPSS 28.0 does not offer the possibility to run automated pooling of MI sets with the GLMM module, nor does it offer output of the covariances of the fixed effects. Therefore, pooling of fixed effects consisting of multiple levels of predictors (e.g., quarter, treatment, format) of the 20 MI sets with a conventional approach was not possible. The chosen strategy to base model selection on GLMM analyses with MI set as additional level is less conservative than a conventional MI strategy, based on Rubin's rule. Thus, after model selection the final model might lead to overestimation of effects and their significance. We therefore analyzed the t-tests of the deviation contrasts with a conventional MI analysis using Rubin's rule for estimating pooled means and s.e.'s (for these the covariances are not required). This guarantees accurate estimates of means, s.e.'s, and contrasts of interest. The final conclusions of the analyses are based on these contrasts, and not on the overall *F*-tests.

## Supplementary material

For supplementary material accompanying this paper visit https://doi.org/10.1017/S0033291722003634. click here to view supplementary material
