# Supplementary material for: Dropout from psychological treatment for borderline personality disorder: a multilevel survival meta-analysis
Source: Psychol Med. 2022 Dec 1;53(3):668–86. doi: 10.1017/S0033291722003634 (PMC9975988; doi:10.1017/S0033291722003634)
Supplement: Supplementary file 1 [file S0033291722003634sup001.pdf]

Overview of appendices

Appendix A. Search terms.

Appendix B. Characteristics of included studies.

Appendix C. References of included studies.

Appendix D. Dropout per quarter by study arm.

Appendix E. Estimation model for missing details of dropout per quarter.

Appendix F. Multiple imputation for missing division of dropout per quarter.

Appendix G. Funnel plots for the 10 treatment categories (complete data set).

Appendix H. Funnel plots for the 10 treatment categories (reduced data set).

Appendix A. Overview of search terms of the database searches.

Pubmed

("Borderline Personality Disorder"[Mesh] OR "borderline personality"[All Fields]) AND (Clinical Trial[ptyp] OR Controlled Clinical Trial[ptyp] OR Randomized Controlled Trial[ptyp])

Embase

(exp borderline personality/ OR borderline personality) AND (clinical trial/de OR controlled clinical trial/de OR randomized controlled trial/de)

Cochrane

"borderline personality"

Psycinfo

'borderline personality'/exp OR 'borderline personality' AND ('clinical trial'/de OR 'controlled clinical trial'/de OR 'randomized controlled trial'/de)

First author and year (study number)	Treatment[1]	N	Proportion Males	Mean Age	Substance Abuse Exclusion	Trial Type[2]	Treatment vs. Study Dropout Distinguished	Intent to Treat Analysis	Setting	Format	Medication dictated by study	Study Length in Quarters	Design Quality Score	Country
Alavi 2021 (571)	DBTmin	55	.25	Missing	Abuse	NRCT	Yes	No	Outpatient	Group	No	1	0.57	Canada
Amianto 2011 (7)	TAU	17	.47	40.10	Dependence	RCT	Yes	No	Outpatient	Individual	No	4	1.11	Italy
	Psychodynamic	18	.56	39.20	No exclusion	RCT	Yes	No	Outpatient	Individual	No	4	1.11	Italy
Arntz 2022 (611)	ST indiv & group	123	.14	33.46	Clin. detox	RCT	Yes	Yes	Outpatient	Combined	No	8	1.78	Australia,
	ST group	125	.13	33.20	Clin. Detox	RCT	Yes	Yes	Outpatient	Group	No	8	1.78	Germany,
	SpecOther (Optimal TAU)	246	.14	33.89	Clin. detox	RCT	Yes	Yes	Outpatient	Combined (majority)	No	8	1.22	Greece, UK, Netherlands[5]
Bales 2012 (9)	MBT	45	.30	30.10	No exclusion	RCT	Yes	Yes	Day treatment	Combined	No	6	1.43	Netherlands
Barnicot 2019 (502)	DBT	58	.28	29.30	No exclusion	RCT	Yes	Yes	Outpatient	Combined	No	4	0.89	
	MBT	32	.28	34.40	No exclusion	RCT	Yes	Yes	Outpatient	Combined	No	4	0.89	
Bateman 1999 (13)	MBT	22	.32	30.30	Abuse	RCT	Yes	No	Day treatment	Combined	No	6	1.33	UK
	TAU	22	.53	33.30	Abuse	RCT	Yes	No	Outpatient	Individual	No	6	1.00	UK
Bateman 2009 (17)	MBT	71	.20	31.30	Clin. detox	RCT	Yes	Yes	Outpatient	Combined	No	6	1.56	UK
	SpecOther (SCM)	63	.21	30.90	Clin. detox	RCT	Yes	Yes	Outpatient	Combined	No	6	1.44	UK
Bellino 2010 (20)	SpecOther (IPT)	27	.30	26.23	Abuse	RCT	Yes	No	Outpatient	Individual	Yes	3	1.11	Italy
Ben-Porath 2004 (24)	DBT	26	.01	35.48	Abuse	OT	Yes	No	Day treatment	Combined	No	2	1.00	USA
Blum 2008 (251)	TAU	72	.14	31.60	Abuse	RCT	Yes	No	Outpatient	Individual	No	2	0.78	USA
Bohus 2000 (37)	DBT	24	.00	28.30	Dependence	OT	Yes	Yes	Inpatient	Combined	No	1	0.38	Germany
Borschmann 2013 (269)	TAU	42	.17	36.10	No exclusion	RCT	Yes	Yes	Outpatient	Individual	No	2	1.11	UK
Bos 2010 (252)	CBT (STEPPS)	45	.17	32.90	No exclusion	RCT	Yes	No	Outpatient	Combined	No	2	1.22	Netherlands
	TAU	38	.11	31.80	No exclusion	RCT	Yes	No	Outpatient	Individual	No	2	0.56	Netherlands
Bozatello 2020 (584)	SpecOther (IPT)	22	.32	35.42	Abuse	RCT	Yes	No	Outpatient	Individual	No	3	1.22	Italy
	TAU (+WL)	21	.35	35.42	Abuse	RCT	Yes	No	Outpatient	Individual	No	3	0.67	Italy
Brassington 2006 (42)	DBT	11	.00	34.30	No exclusion	OT	No	No	Outpatient	Combined	No	2	1.25	New Zealand
Brown 2004 (43)	CBT	32	.13	29.00	Clin. detox	OT	Yes	Yes	Outpatient	Individual	No	4	1.50	USA
Carmona I Farres 2019 (572)	DBTmin (Interpers Skills)	32	.16	33.75	Dependence	RCT	Yes	Yes	Outpatient	Combined	No	1	1.44	Spain
	DBTmin (Mindfulness)	33	.06	31.03	Dependence	RCT	Yes	Yes	Outpatient	Combined	No	1	1.44	Spain
Carter 2010 (45)	DBT	38	.00	24.50	Clin. detox	RCT	No	Yes	Outpatient	Combined	No	2	1.33	Australia
	TAU + WL	35	.00	24.70	Clin. detox	RCT	No	Yes	Outpatient	Individual	No	2	0.89	Australia
Chalker 2015 (423)	DBT	63	.27	37.20	No exclusion	OT	Yes	Yes	Outpatient	Combined	No	4	1.13	USA
Chanen 2021 (565)	Mixed (CAT + HYPE)	24	0.25	21.35	No exclusion	RCT	Yes	Yes	Outpatient	Individual	No	5	1.56	Australia

	Mixed (HYPE+ befriending)	27	0.30	20.83	No exclusion	RCT	Yes	Yes	Outpatient	Individual	No	5	1.56	Australia
	Mixed (YMHS + befriending)	25	0.20	21.31	No exclusion	RCT	Yes	Yes	Outpatient	Individual	No	5	1.33	Australia
Clarkin 2001 (52)	TFP	23	.00	32.70	No exclusion	OT	Yes	No	Outpatient	Individual	No	4	1.25	USA
Clarkin 2007 (53)	TFP	31	.08	30.90	Dependence	RCT	No	No	Outpatient	Individual	No	4	1.44	USA
	DBT	30	.08	30.90	Dependence	RCT	No	No	Outpatient	Combined	No	4	1.44	USA
	Psychodynamic	29	.08	30.90	Dependence	RCT	No	No	Outpatient	Individual	No	4	1.44	USA
Comtois 2007 (55)	DBT	38	.04	34.00	Dependence	OT	No	No	Outpatient	Combined	No	4	0.88	USA
Comtois 2010 (51)[4]	DBT[4]	85	Missing	Missing	Dependence[4]	OT	No	No	Outpatient	Combined	No	4	0.63	USA
Cottraux 2009 (57)	CBT	33	.27	34.30	Dependence	RCT	No	Yes	Outpatient	Individual	No	4	1.56	France
	SpecOther (CCT)	32	.19	32.60	Dependence	RCT	No	Yes	Outpatient	Individual	No	4	1.56	France
Davidson 2006 (61)	CBT (+ TAU)	54	.17	32.60	Dependence	RCT	No	Yes	Outpatient	Individual	No	4	1.89	UK
	TAU	52	.15	31.40	Dependence	RCT	No	Yes	Outpatient	Individual	No	4	1.22	UK
Del Pozo 2018 (405)	Mixed	269	.15	35.00	Dependence	OT	Yes	Yes	Inpatient	Combined	No	1	0.86	Germany
Dickhaut 2014 (220)	ST	18	.00	28.50	Clin. detox	OT	Yes	Yes	Outpatient	Combined	No	10	1.25	Netherlands
Doering 2010 (67)	TFP	52	.00	27.46	Dependence	RCT	Yes	Yes	Outpatient	Individual	No	4	2.00	Austria & Germany
	CTBE	52	.00	27.19	Dependence	RCT	Yes	Yes	Outpatient	Individual	No	4	1.67	Austria & Germany
Farrell 2009 (70)	ST (+ TAU)	16	.00	35.30	No exclusion	RCT	Yes	No	Outpatient	Combined	No	3	1.11	USA
	TAU	16	.00	35.90	No exclusion	RCT	Yes	No	Outpatient	Individual	No	3	0.67	USA
Fassbinder 2016 (413)	ST	10	.00	35.00	Clin. detox	OT	Yes	Yes	Outpatient	Combined	No	4	1.25	Germany
Feigenbaum 2012 (73)	DBT	25	.28	35.40	Clin. detox	RCT	Yes	Yes	Outpatient	Combined	No	4	1.56	UK
	TAU	16	.25	34.60	Clin. detox	RCT	Yes	Yes	Outpatient	Individual	No	4	1.00	UK
Fitzpatrick 2020 (553)	DBT	101	.24	29.54	No exclusion	OT	Yes	Yes	Outpatient	Combined	No	2	1.14	USA
Friedrich 2003 (223)	DBT	33	.09	33.40	No exclusion	OT	Yes	No	Outpatient	Combined	No	4	0.43	Germany
Gaglia 2013 (297) (DBT-cases from Priebe 2012 excluded)	DBT	62	.11	32.01	No exclusion	OT	Yes	Yes	Outpatient	Combined	No	4	1.00	UK
Giesen-Bloo 2006 (75)	ST	45	.09	31.70	Clin. detox	OT	Yes	Yes	Outpatient	Individual	No	12	1.56	Netherlands
	TFP	43	.05	29.40	Clin. detox	OT	Yes	Yes	Outpatient	Individual	No	12	1.56	Netherlands
Gratz 2006 (82)	Mixed	36	.22	28.64	Unclear	OT	Yes	No	Day treatment	Combined	No	1	0.63	USA
Harley 2007 (89)	DBT	10	.43	39.00	No exclusion	OT	Yes	No	Outpatient	Combined	No	3	0.71	USA
	DBT min (+TAU)	39	.43	39.00	No exclusion	OT	Yes	No	Outpatient	Combined	No	3	0.71	USA
Hilden 2020 (569)	ST (+TAU)	25	.08	31.00	Abuse	RCT	Yes	Yes	Outpatient	Combined	No	2	1.88	Finland
	TAU	12	.00	27.00	Abuse	RCT	Yes	Yes	Outpatient	Individual	No	2	1.13	Finland
Höschel 2006 (95)	DBT	26	.12	28.25	Dependence	OT	No	No	Inpatient	Combined	No	1	0.29	Germany
Jacob 2018 (444)	ST (+app)	14	.15	28.40	Clin. Detox	OT	Yes	Yes	Outpatient	Individual	No	4	0.50	Germany
Jimenez 2022 (628)	DBTmin	229	.10	31.10	Dependence	NRCT	Yes	Yes	Outpatient	Group	No	2	0.43	Mexico
Jorgensen 2013 (99)	MBT	58	.04	29.20	Clin. detox	RCT	Yes	Yes	Outpatient	Combined	No	8	1.44	Denmark
	Psychodynamic	27	.05	29.00	Clin. detox	RCT	Yes	Yes	Outpatient	Group	No	8	1.22	Denmark

Karterud 1992 (105)	SpecOther (Therapeutic Community)	34	Missing	Missing	No exclusion	OT	No	Yes	Day treatment	Combined	No	3	0.57	Norway
Karterud 2003 (104)	Mixed	275	Missing	Missing	No exclusion	OT	Yes	Yes	Day treatment	Combined	No	2	0.71	Norway
Kellett 2013 (281)	SpecOther (CAT)	19	.18	29.99	No exclusion	OT	Yes	No	Outpatient	Individual	No	2	1.00	USA
Kells 2020 (561)	DBTmin	41	.20	35.61	No exclusion	OT	Yes	Yes	Outpatient	Group	No	2	1.14	Ireland
Kleindienst 2011 (38)	DBT	74	.00	27.60	Abuse	NRCT	No	No	Inpatient	Combined	No	1	0.88	Germany
	TAU + WL	20	.00	29.50	Abuse	NRCT	No	No	In / outpatient[3]	Combined	No	1	0.50	Germany
Koons 2001 (111)	DBT	14	.00	34.50	Dependence	RCT	Yes	No	Outpatient	Combined	No	2	1.56	USA
	TAU	14	.00	35.40	Dependence	RCT	Yes	No	Outpatient	Combined	No	2	1.00	USA
Kramer 2016 (432)	DBTmin (+TAU)	21	.05	35.10	No exclusion	RCT	Yes	Yes	Outpatient	Group	No	2	1.44	Switzerland
	TAU	20	.20	33.60	No exclusion	RCT	Yes	Yes	Outpatient	Individual	No	2	1.00	Switzerland
Kröger 2006 (118)	DBT	50	.12	30.50	Clin. detox	OT	No	No	Inpatient	Combined	No	1	0.88	Germany
Kröger 2013 (284)	DBT	1423	.32	32.00	Clin. detox	OT	Yes	Yes	Inpatient	Combined	No	1	1.43	Germany
Kröger 2014 (226)	DBT	541	.10	29.00	No exclusion	OT	Yes	No	Inpatient	Combined	No	1	1.57	Germany
Kröger 2015 (232)	Psychodynamic	269	.16	35.00	Clin. detox	OT	Yes	Yes	Inpatient	Combined	No	1	1.43	Germany
Lana 2015 (415)	Mixed	40	.33	33.00	Dependence	OT	No	Yes	Day Treatment	Combined	No	2	1.14	Spain
Laurenssen 2018 (412)	MBT	54	.22	34.00	Clin detox	RCT	Yes	Yes	Day Treatment	Combined	No	6	1.33	Netherlands
	TAU	41	.18	34.00	Clin detox	RCT	Yes	Yes	Outpatient	Combined	No	6	1.11	Netherlands
Leerer 1997 (121)	DBTmin	14	.00	35.00	Clin. detox	OT	Yes	Yes	Inpatient	Combined	No	4	0.88	USA
Leppänen 2016 (422)	Mixed	24	.16	31.90	Abuse	RCT	Yes	No	Outpatient	Combined	No	4	1.00	Finland
	TAU	47	.13	32.30	Abuse	RCT	Yes	No	Outpatient	Individual	No	4	0.78	Finland
Lin 2019 (603)	DBTmin	42	.10	20.40	Abuse	RCT	Yes	Yes	Outpatient	Group	No	1	1.56	China
	CBT (group)	40	.15	20.47	Abuse	RCT	Yes	Yes	Outpatient	Group	No	1	1.56	China
Linehan 1991 (126)	DBT	24	.00	31.50	Dependence	RCT	No	Yes	Outpatient	Combined	No	4	1.67	USA
	TAU	22	.00	31.50	Dependence	RCT	No	Yes	Outpatient	Individual	No	4	1.11	USA
Linehan 2006 (130)	DBT	52	.00	29.00	No exclusion	RCT	Yes	Yes	Outpatient	Combined	No	4	1.89	USA
	CTBE	49	.00	29.63	No exclusion	RCT	Yes	Yes	Outpatient	Individual	No	4	1.56	USA
Linehan 2008 (129)	DBT	24	.00	36.80	Dependence	OT	Yes	Yes	Outpatient	Combined	Yes	2	1.67	USA
Linehan 2015 (228)	DBT	33	.00	31.10	No exclusion	RCT	Yes	Yes	Outpatient	Combined	No	4	1.89	USA
	DBTmin (individual) (+ TAU)	33	.00	30.10	No exclusion	RCT	Yes	Yes	Outpatient	Combined	No	4	1.89	USA
	DBTmin (group) (+ TAU)	33	.00	29.80	No exclusion	RCT	Yes	Yes	Outpatient	Combined	No	4	1.89	USA
Löf 2018 (411)	MBT	75	.11	30.40	Clin detox	OT	Yes	Yes	Outpatient	Combined	No	6	1.29	Sweden
Löffler-Stastka 2006 (135)	Psychodynamic	20	.50	38.30	Clin. detox	OT	Yes	Yes	Inpatient	Combined	No	1	0.57	Austria
Lyng 2019 (520)	DBTyouth	24	.17	20.50	Dependence	NRCT	No	No	Outpatient	Combined	No	4	.63	Ireland

	DBT	13	.31	20.50	Dependence	NRCT	No	No	Outpatient	Combined	No	4	.63	Ireland
Majdara 2021 (564)	SpecOther (DDP)	15	.33	28.08	No exclusion	RCT	Yes	Yes	Outpatient	Individual	No	4	1.44	Iran
	TAU (EUC)	15	.47	26.60	No exclusion	RCT	Yes	Yes	Outpatient	Group	No	4	1.00	Iran
Markovitz 2007 (138)	SpecOther (IPT)	11	.13	34.00	No exclusion	OT	Yes	No	Outpatient	Individual	No	3	1.00	USA
McMain 2009 (143)	DBT	90	.10	29.40	Dependence	RCT	Yes	Yes	Outpatient	Combined	No	4	2.00	Canada
	SpecOther (GPM)	90	.18	31.30	Dependence	RCT	Yes	Yes	Outpatient	Individual	No	4	1.89	Canada
McMain 2017 (408)	DBTmin	42	.17	27.30	No exclusion	RCT	Yes	Yes	Outpatient	Group	No	2	1.67	Canada
Meares 1999 (147)	Psychodynamic	48	Missing	29.40	Unclear	NRCT	No	No	Outpatient	Individual	No	4	1.00	Australia
Moen 2012 (150)	DBT	18	.20	35.50	Dependence	OT	No	No	Outpatient	Combined	Yes	1	1.67	USA
Morey 2010 (151)	CBT (MACT)	8	.25	29.63	No exclusion	RCT	Yes	Yes	Outpatient	Individual	No	1	1.33	USA
	CBT (MACT+TA)	8	.13	32.50	No exclusion	RCT	Yes	Yes	Outpatient	Individual	No	1	1.33	USA
Nadort 2009 (153)	ST	62	.03	31.97	Clin. detox	RCT	Yes	Yes	Outpatient	Individual	No	6	1.89	Netherlands
Nordahl 2005 (157)	ST	6	.00	25.60	No exclusion	OT	Yes	No	Outpatient	Individual	No	12	0.71	Netherlands
Nordahl 2019 (518)	CBT	12	.17	23.10	Dependence	OT	Yes	Yes	Outpatient	Individual	No	4	1.14	Norway
Nyseater 2010 (158)	Psychodynamic	32	.19	28.90	Clin. detox	OT	Yes	Yes	Outpatient	Individual	No	6	0.88	Norway
Paret 2021 (575)	DBT	22	.00	21.05	Unclear	OT	Yes	Yes	Inpatient	Combined	No	1	0.43	Germany
Pascienzy 2011 (163)	DBT	43	.05	33.58	No exclusion	NRCT	No	No	Outpatient	Combined	No	2	0.88	Australia
	TAU + WL	47	.08	33.17	No exclusion	NRCT	No	No	Outpatient	Individual	No	2	0.25	Australia
Prendergast 2007 (224)	DBT	16	.00	36.35	No exclusion	OT	Yes	No	Outpatient	Combined	No	2	0.88	Australia
Priebe 2012 (169)	DBT	40	.13	33.00	No exclusion	RCT	Yes	Yes	Outpatient	Combined	No	4	1.44	UK
	TAU	40	.13	31.30	No exclusion	RCT	Yes	Yes	Outpatient	Combined	No	4	1.00	UK
Reiss 2014 (229)	ST	42	.02	36.40	No exclusion	OT	Yes	No	Inpatient	Combined	No	2	1.14	USA
Reiss 2014 (230)	ST	37	.11	31.60	No exclusion	OT	Yes	No	Inpatient	Combined	No	1	1.14	USA
Reiss 2014 (231)	ST	16	.00	25.50	No exclusion	OT	Yes	No	Inpatient	Combined	No	1	1.00	Germany
Rizvi 2017 (407)	DBT	50	.20	29.50	No exclusion	OT	Yes	Yes	Outpatient	Combined	No	2	1.13	USA
Rüsch 2008 (173)	DBT	60	.00	27.80	No exclusion	OT	Yes	Yes	Inpatient	Combined	No	1	0.43	Germany
Ryle 2000 (174)	SpecOther (CAT)	37	.41	34.30	No exclusion	OT	Yes	No	Outpatient	Individual	No	2	1.00	UK
Sachdeva 2013 (292)	DBT	25	.16	36.60	No exclusion	NRCT	Yes	Yes	Outpatient	Combined	No	4	1.13	USA
(also Gregory 2016)	SpecOther (DDP)	27	.15	28.00	No exclusion	NRCT	Yes	Yes	Outpatient	Individual	No	4	1.13	USA
	TAU	16	.31	29.30	No exclusion	NRCT	Yes	Yes	Outpatient	Combined	No	4	0.50	USA
Sachse 2011 (176)	CBT (MBCT) (+TAU)	26	.14	38.96	Clin. detox	OT	Yes	Yes	Outpatient	Group	No	1	1.00	UK
Sandell 1993 (178)	Psychodynamic	132	.49	35.05	No exclusion	OT	Yes	No	Day treatment	Combined	No	2	0.29	Sweden
Simpson 2004 (182)	DBT	25	.00	35.27	Dependence	RCT	Yes	No	Outpatient	Combined	Yes	1	1.33	USA
Sinnaeve 2018 (410)	DBT(outpatient)	42	.05	25.60	Clin. detox	RCT	Yes	No	Outpatient	Combined	No	4	1.56	Netherlands
	DBT(inpatient)	42	.05	26.20	Clin. detox	RCT	Yes	No	Inpatient	Combined	No	3	1.56	Netherlands
Smits 2019 (400)	MBT(outpatient)	44	.20	29.90	No exclusion	RCT	No	Yes	Outpatient	Combined	No	6	1.50	Netherlands
	MBT(day treatment)	47	.16	31.40	No exclusion	RCT	No	Yes	Inpatient	Combined	No	6	1.50	Netherlands
Soler 2005 (185)	DBTmin	60	.13	26.95	Dependence	RCT	No	Yes	Outpatient	Group	No	1	1.00	Spain
Soler 2009 (186)	DBTmin	30	.22	28.45	Dependence	RCT	Yes	Yes	Outpatient	Group	No	1	1.44	Spain
	TAU	30	.13	29.97	Dependence	RCT	Yes	Yes	Outpatient	Group	No	1	1.00	Spain

Soler 2012	TAU	19	.21	31.78	Dependence	NRCT	Yes	Yes	Outpatient	Individual	Yes	1	0.75	Spain
Sollberger 2014 (233)	Mixed (TFP & DBT)	34	.19	26.70	Abuse	NRCT	Yes	No	Inpatient	Combined	No	1	1.00	Switzerland
	TAU	21	.25	29.40	Abuse	NRCT	Yes	No	Inpatient	Combined	No	1	0.43	Switzerland
Stanley 2007 (189)	DBT	20	.15	32.20	No exclusion	OT	Yes	Yes	Outpatient	Combined	No	2	0.75	USA
Stiglmayr 2014 (222)	DBT	78	.09	30.10	Dependence	OT	Yes	No	Outpatient	Combined	No	4	1.50	Germany
Thormahlen 2003 (299)	Psychodynamic	12	Missing	34.50	Abuse	RCT	Yes	No	Outpatient	Individual	No	4	1.14	Sweden
Turner 2000 (196)	DBTmin	12	.21	22.00	Clin. detox	RCT	Yes	Yes	Outpatient	Combined	No	4	1.67	USA
	SpecOther (CCT)	12	.21	22.00	Clin. detox	RCT	Yes	Yes	Outpatient	Combined	No	4	1.67	USA
Vaglum 1990 (218)	SpecOther (TherCom)	44	Missing	Missing	No exclusion	OT	Yes	No	Day treatment	Combined	No	2	0.25	Norway
Vaillancourt 2012 (430)	DBT	241	.04	35.90	Dependence	OT	No	No	Day treatment	Combined	No	4	0.75	USA
Van den Bosch 2013 (202)	DBT	39	.00	26.00	Clin. detox	OT	Yes	Yes	Day treatment	Combined	No	1	1.13	Netherlands
Verheul 2003 (206)	DBT	31	.00	35.10	No exclusion	RCT	Yes	No	Outpatient	Combined	No	4	1.44	Netherlands
	TAU	33	.00	34.70	No exclusion	RCT	Yes	No	Outpatient	Individual	No	4	0.89	Netherlands
Walton 2020 (540)	DBT	83	.23	25.80	Dependence	RCT	Yes	Yes	Outpatient	Combined	No	5	1.78	Australia
	Psychodynamic	83	.22	27.30	Dependence	RCT	Yes	Yes	Outpatient	Individual	No	5	1.78	Australia
Wilberg 1998a (210)	Psychodynamic	53	.23	31.00	No exclusion	OT	No	No	Day treatment	Combined	No	3	0.50	Norway
Wilberg 1998b (219)	Mixed (Psydyn + CBT)	70	Missing	Missing	No exclusion	OT	Yes	Yes	Day treatment	Group	No	2	0.63	Norway
Wildgoose 2001 (211)	SpecOther (CAT)	5	.40	39.40	Abuse	OT	Yes	No	Outpatient	Individual	No	2	1.14	UK
Yeomans 1993 (213)	TFP	14	Missing	Missing	Unclear	OT	No	No	Outpatient	Individual	No	8	1.29	USA
Zinkler 2007 (215)	DBT	49	.12	33.21	No exclusion	OT	Yes	No	Outpatient	Combined	No	4	1.14	UK

Note.
[1] CAT = Cognitive Analytic Therapy; CCT = Client Centered Therapy; CTBE = Community Treatment by Experts; DBT = Dialectical Behavior Therapy; DDP = Dynamic Deconstructive Psychotherapy; GPM = General Psychiatric Management; MBT = Mentalization Based Treatment; SCM = Structured Clinical Management; ST = Schema Therapy; SpecOther = specified other treatment; PsyDyn = Psychodynamic Therapy; SpecOther = Specified Other; TAU = Treatment as Usual; TFP = Transference Focused Psychotherapy.
[2] RCT = Randomized Clinical/Controlled Trial; NRCT = NonRandomized Controlled Trial; OT = Open Trial (includes uncontrolled cohort studies and case series studies).
[3] Coded as inpatient given the high use of inpatient treatment.
[4] Only the standard one-year DBT program data were used, not the DBT-ACES program offered as next step to a subsample of those that completed standard DBT. See Comtois et al (2007) for in/exclusion criteria of the site's DBT program.
[5] As only 6.7% of participants came from Australia and all others from Europe, the study was coded as country group "Europe".

Appendix C. References of studies included in the analysis.

Alavi, N., Stephenson, C., & Rivera, M. (2021). Effectiveness of delivering dialectical behavioral therapy techniques by email in patients with borderline personality disorder: Nonrandomized controlled trial. *JMIR Mental Health, 8*(4), e27308–e27308.

Amianto, F., Ferrero, A., Pierò, A., Cairo, E., Rocca, G., Simonelli, B., Fassina, S., Abbate-Daga, G. & Fassino, S. (2011). Supervised team management, with or without structured psychotherapy, in heavy users of a mental health service with borderline personality disorder: A two-year follow-up preliminary randomized study. *BMC Psychiatry*, *11*(1), 181.

Arntz, A., Jacob, G. A., Lee, C. W., Brand-de Wilde, O. M., Fassbinder, E., Harper, R. P., Lavender, A., Lockwood, G., Malogiannis, I. A., Ruths, F. A., Schweiger, U., Shaw, I. A., Zarbock, G., & Farrell, J. M. (2022). Effectiveness of predominantly group schema therapy and combined individual and group schema therapy for borderline personality disorder: A randomized clinical trial. *JAMA Psychiatry (Chicago, Ill.), 79*(4), 287–299.

Bales, D., van Beek, N., Smits, M., Willemsen, S., Busschbach, J. J., Verheul, R., & Andrea, H. (2012). Treatment outcome of 18-month, day hospital mentalization-based treatment (MBT) in patients with severe borderline personality disorder in the Netherlands. *Journal of Personality Disorders*, *26*(4), 568-582.

Barnicot, K., & Crawford, M. (2019). Dialectical behaviour therapy v. mentalisation-based therapy for borderline personality disorder. *Psychological Medicine, 49*(12), 2060–2068.

Bateman, A., & Fonagy, P. (1999). Effectiveness of partial hospitalization in the treatment of borderline personality disorder: a randomized controlled trial. *American Journal of Psychiatry*, *156*(10), 1563-1569. [see also Bateman & Fonagy, 2001]

Bateman, A., & Fonagy, P. (2001). Treatment of borderline personality disorder with psychoanalytically oriented partial hospitalization: an 18-month follow-up. *American Journal of Psychiatry, 158(1),* 36-42. [see Bateman & Fonagy, 1999]

Bateman, A., & Fonagy, P. (2009). Randomized controlled trial of outpatient mentalization-based treatment versus structured clinical management for borderline personality disorder. *The American Journal of Psychiatry*, *166*(12), 1355-1364.

Bellino, S., Rinaldi, C., & Bogetto, F. (2010). Adaptation of interpersonal psychotherapy to borderline personality disorder: a comparison of combined therapy and single pharmacotherapy. *Canadian Journal of Psychiatry*, *55*(2), 74.

Ben-Porath, D. D., Peterson, G. A., & Smee, J. (2004). Treatment of individuals with borderline personality disorder using dialectical behavior therapy in a community mental health setting: Clinical application and a preliminary investigation. *Cognitive and Behavioral Practice*, *11*(4), 424-434.

Blum, N., St. John, D., Pfohl, B., Stuart, S., McCormick, B., Allen, J., ... & Black, D. W. (2008). Systems Training for Emotional Predictability and Problem Solving (STEPPS) for outpatients with borderline personality disorder: a randomized controlled trial and 1-year follow-up. *American Journal of Psychiatry, 165(4),* 468-478.

Bohus, M., Haaf, B., Stiglmayr, C., Pohl, U., Boëhme, R., & Linehan, M. (2000). Evaluation of inpatient dialectical-behavioral therapy for borderline personality disorder—a prospective study. *Behaviour research and therapy*,*38*(9), 875-887.

Bohus, M., Haaf, B., Simms, T., Limberger, M. F., Schmahl, C., Unckel, C., Lieb, K. & Linehan, M. M. (2004). Effectiveness of inpatient dialectical behavioral therapy for borderline personality disorder: a controlled trial. *Behaviour Research and Therapy, 42(5),* 487-499. [see also Kleindienst et al., 2011]

Borschmann, R., Barrett, B., Hellier, J. M., Byford, S., Henderson, C., Rose, D., ... & Hogg, J. (2013). Joint crisis plans for people with borderline personality disorder: feasibility and outcomes in a randomised controlled trial. *The British Journal of Psychiatry, 202(5),* 357-364.

Bos, E. H., van Wel, E. B., Appelo, M. T., & Verbraak, M. J. (2010). A randomized controlled trial of a Dutch version of systems training for emotional predictability and problem solving for borderline personality disorder. *The Journal of Nervous and Mental Disease, 198(4),* 299-304.

Bozzatello, P., & Bellino, S. (2020). Interpersonal psychotherapy as a single treatment for borderline personality disorder: A pilot randomized-controlled study. *Frontiers in Psychiatry, 11,* 578910–578910.

Brassington, J., & Krawitz, R. (2006). Australasian dialectical behaviour therapy pilot outcome study: effectiveness, utility and feasibility. *Australasian Psychiatry*, *14*(3), 313-318.

Brown, G. K., Newman, C. F., Charlesworth, S. E., Crits-Christoph, P., & Beck, A. T. (2004). An open clinical trial of cognitive therapy for borderline personality disorder. *Journal of Personality Disorders*, *18*(3: Special issue), 257-271.

Carmona i Farrés, C., Elices, M., Soler, J., Domínguez-Clavé, E., Martín-Blanco, A., Pomarol-Clotet, E., Salvador, R., Martinez-Horta, S., & Pascual, J. C. (2019). Effects of mindfulness training on the default mode network in borderline personality disorder. *Clinical Psychology and Psychotherapy, 26*(5), 562–571.

Carter, G. L., Willcox, C. H., Lewin, T. J., Conrad, A. M., & Bendit, N. (2010). Hunter DBT project: randomized controlled trial of dialectical behaviour therapy in women with borderline personality disorder. *Australian and New Zealand Journal of Psychiatry*, *44*(2), 162-173.

Chalker, S. A., Carmel, A., Atkins, D. C., Landes, S. J., Kerbrat, A. H., & Comtois, K. A. (2015). Examining challenging behaviors of clients with borderline personality disorder. *Behaviour Research and Therapy, 75,* 11–19.

Chanen, A. M., Betts, J. K., Jackson, H., Cotton, S. M., Gleeson, J., Davey, C. G., Thompson, K., Perera, S., Rayner, V., Andrewes, H., & McCutcheon, L. (2022). Effect of 3 forms of early intervention for young people with borderline personality disorder: The MOBY randomized clinical trial. *JAMA Psychiatry (Chicago, Ill.), 79*(2), 109–119.

Clarkin, J. F., Foelsch, P. A., Levy, K. N., Hull, J. W., Delaney, J. C., & Kernberg, O. F. (2001). The development of a psychodynamic treatment for patients with borderline personality disorder: a preliminary study of behavioral change. *Journal of Personality Disorders*, *15*(6), 487-495. [see also Clarkin et al., 2005]

Clarkin, J. F., Levy, K. N., Lenzenweger, M. F., & Kernberg, O. F. (2007). Evaluating three treatments for borderline personality disorder: a multiwave study. *American Journal of Psychiatry*, *164*(6), 922-928.

Clarkin, J. F., Levy, K. N., & Schiavi, J. M. (2005). Transference focused psychotherapy: development of a psychodynamic treatment for severe personality disorders. *Clinical Neuroscience Research, 4(5),* 379-386. [see Clarkin et al., 2001]

Comtois, K. A., Elwood, L., Holdcraft, L. C., Smith, W. R., & Simpson, T. L. (2007). Effectiveness of dialectical behavior therapy in a community mental health center. *Cognitive and Behavioral Practice*, *14*(4), 406-414.

Comtois, K. A., Holland Kerbrat, A., Atkins, D. C., Harned, M. S., & Elwood, L. (2010). Recovery from disability for individuals with borderline personality disorder: A feasibility trial of DBT-ACES. *Psychiatric Services (Washington, D.C.), 61*(11), 1106–1111.

Cottraux, J., Boutitie, F., Milliery, M., Genouihlac, V., Yao, S. N., Mollard, E., Bonasse, F., Gaillard, S., Djamoussain, D., de Mey Guillard, C., Culem, A. & Gueyffier, F. (2009). Cognitive therapy versus Rogerian supportive therapy in borderline personality disorder. *Psychotherapy and psychosomatics*, *78*(5), 307-316.

Davidson, K., Norrie, J., Tyrer, P., Gumley, A., Tata, P., Murray, H., & Palmer, S. (2006). The effectiveness of cognitive behavior therapy for borderline personality disorder: results from the borderline personality disorder study of cognitive therapy (BOSCOT) trial. *Journal of personality disorders*, *20*(5), 450.

del Pozo, M. A., Kliem, S., & Mestel, R. (2018). Stationäre psychodynamisch orientierte multimodale Therapie von Patienten mit Borderline-Persönlichkeitsstörung: Wirksamkeit, Response und Drop-out. *Psychotherapie, Psychosomatik, Medizinische Psychologie, 68*(2), 82–90.

Dickhaut, V., & Arntz, A. (2014). Combined group and individual schema therapy for borderline personality disorder: a pilot study. *Journal of behavior therapy and experimental psychiatry*, *45*(2), 242-251.

Doering, S., Hörz, S., Rentrop, M., Fischer-Kern, M., Schuster, P., Benecke, C., Buchheim, A., Marius, P. & Buchheim, P. (2010). Transference-focused psychotherapy v. treatment by community psychotherapists for borderline personality disorder: randomised controlled trial. *The British Journal of Psychiatry*, *196*(5), 389-395.

Farrell, J. M., Shaw, I. A., & Webber, M. A. (2009). A schema-focused approach to group psychotherapy for outpatients with borderline personality disorder: a randomized controlled trial. *Journal of Behavior Therapy and Experimental Psychiatry*, *40*(2), 317-328.

Fassbinder, E., Rudolf, S., Bussiek, A., Kröger, C., Arnold, R., Greggersen, W., Hüppe. M., Sipos, V. & Schweiger, U. (2007). Effektivität der dialektischen Verhaltenstherapie bei Patienten mit Borderline-Persönlichkeitsstörung im Langzeitverlauf–Eine 30-Monats-Katamnese nach stationärer Behandlung. *Psychotherapie, Psychosomatik, Medizinische Psychologie, 57*, 161-169. [see Kröger et al., 2006]

Fassbinder, E., Schuetze, M., Kranich, A., Sipos, V., Hohagen, F., Shaw, I., Farrell, J., Arntz, A., & Schweiger, U. (2016). Feasibility of group schema therapy for outpatients with severe borderline personality disorder in Germany: A pilot study with three year follow-up. *Frontiers in Psychology, 7,* 1851–1851.

Feigenbaum, J. D., Fonagy, P., Pilling, S., Jones, A., Wildgoose, A., & Bebbington, P. E. (2012). A real-world study of the effectiveness of DBT in the UK National Health Service. *British Journal of Clinical Psychology*, *51*(2), 121-141.

Fitzpatrick, S., Bailey, K., & Rizvi, S. L. (2020). Changes in emotions over the course of dialectical behavior therapy and the moderating role of depression, anxiety, and posttraumatic stress disorder. *Behavior Therapy, 51*(6), 946–957.

Friedrich, J., Gunia, H., & Huppertz, M. (2003). Evaluation eines ambulanten Netzwerks für Dialektisch Behaviorale Therapie. *Verhaltenstherapie & Verhaltensmedizin*.

Gaglia, A., Essletzbichler, J., Barnicot, K., Bhatti, N., & Priebe, S. (2013). Dropping out of dialectical behaviour therapy in the NHS: the role of care coordination. *The Psychiatrist Online, 37(8),* 267-271.

Giesen-Bloo, J., Van Dyck, R., Spinhoven, P., Van Tilburg, W., Dirksen, C., Van Asselt, T., Kremers, I., Nadort, N. & Arntz, A. (2006). Outpatient psychotherapy for borderline personality disorder: randomized trial of schema-focused therapy vs transference-focused psychotherapy. *Archives of General Psychiatry*, *63*(6), 649-658.

Gratz, K. L., Lacroce, D. M., & Gunderson, J. G (2006). Measuring changes in symptoms relevant to borderline personality disorder following short-term treatment across partial hospital and intensive outpatient levels of care. *Journal of Psychiatric Practice, 12(3)*, 153-159.

Gregory, R. J., & Sachdeva, S. (2016). Naturalistic Outcomes of Evidence-Based Therapies for Borderline Personality Disorder at a Medical University Clinic. *American Journal of Psychotherapy, 70(2),* 167-184. [see Sachdeva et al., 2013]

Harley, R. M., Baity, M. R., Blais, M. A., & Jacobo, M. C. (2007). Use of dialectical behavior therapy skills training for borderline personality disorder in a naturalistic setting. *Psychotherapy Research*, *17*(3), 351-358.

Hilden, H.-M., Rosenström, T., Karila, I., Elokorpi, A., Torpo, M., Arajärvi, R., & Isometsä, E. (2021). Effectiveness of brief schema group therapy for borderline personality disorder symptoms: A randomized pilot study. *Nordic Journal of Psychiatry, 75*(3), 176–185.

Höschel, K. (2006). Dialektisch Behaviorale Therapie der Borderline Persönlichkeitsstörung in der Regelversorgung—Das Saarbrücker DBT Modell [Dialectic behavioral therapy for borderline personality disorder in standard medical care: The Saarbrücken Treatment Program]. *Verhaltenstherapie, 16*, 17–24.

Jacob, G. A., Hauer, A., Köhne, S., Assmann, N., Schaich, A., Schweiger, U., & Fassbinder, E. (2018). A schema therapy-based eHealth program for patients with borderline personality disorder (priovi): Naturalistic single-arm observational study. *JMIR Mental Health, 5*(4), e10983–e10983.

Jiménez, S., Angeles-Valdez, D., Rodríguez-Delgado, A., Fresán, A., Miranda, E., Alcalá-Lozano, R., Duque-Alarcón, X., Arango de Montis, I., & Garza-Villarreal, E. A. (2022). Machine learning detects predictors of symptom severity and impulsivity

after dialectical behavior therapy skills training group in borderline personality disorder. *Journal of Psychiatric Research, 151*, 42–49.

Jørgensen, C. R., Freund, C., Bøye, R., Jordet, H., Andersen, D., & Kjølbye, M. (2013). Outcome of mentalization-based and supportive psychotherapy in patients with borderline personality disorder: a randomized trial. *Acta Psychiatrica Scandinavica*, *127*(4), 305-317.

Karterud, S., Vaglum, S., Friis, S., Irion, T., Johns, S., & Vaglum, P. (1992). Day hospital therapeutic community treatment for patients with personality disorders: An empirical evaluation of the containment function. *The Journal of nervous and mental disease*, *180*(4), 238-243.

Karterud, S., Pedersen, G., Bjordal, E., Brabrand, J., Friis, S., Haaseth, Ø., Haavaldsen, G., Irion, T., Leirvag, H., Torum. E. & Urnes, Ø. (2003). Day treatment of patients with personality disorders: experiences from a Norwegian treatment research network. *Journal of Personality Disorders*, *17*(3), 243-262.

Kellett, S., Bennett, D., Ryle, T., & Thake, A. (2013). Cognitive analytic therapy for borderline personality disorder: therapist competence and therapeutic effectiveness in routine practice. *Clinical Psychology & Psychotherapy, 20(3),* 216-225.

Kells, M., Joyce, M., Flynn, D., Spillane, A., & Hayes, A. (2020). Dialectical behaviour therapy skills reconsidered: applying skills training to emotionally dysregulated individuals who do not engage in suicidal and self-harming behaviours. *Borderline Personality Disorder and Emotion Dysregulation, 7*(1), 3–3.

Kleindienst, N., Limberger, M. F., Ebner-Priemer, U. W., Keibel-Mauchnik, J., Dyer, A., Berger, M.…Bohus, M. (2011). Dissociation predicts poor response to dialectical behavioral therapy in female patients with borderline personality disorder. *Journal of Personality Disorders, 25(4)*, 432-447. [see also Bohus et al., 2004]

Koons, C. R., Robins, C. J., Tweed, J. L., Lynch, T. R., Gonzalez, A. M., Morse, J. Q., ... & Bastian, L. A. (2001). Efficacy of dialectical behavior therapy in women veterans with borderline personality disorder. *Behavior Therapy*, *32*(2), 371-390.

Kramer, U., Pascual-Leone, A., Berthoud, L., de Roten, Y., Marquet, P., Kolly, S., Despland, J.-N., & Page, D. (2016). Assertive anger mediates effects of dialectical behaviour-informed skills training for borderline personality disorder: A randomized controlled trial. *Clinical Psychology and Psychotherapy, 23*(3), 189–202.

Kröger, C., Schweiger, U., Sipos, V., Arnold, R., Kahl, K. G., Schunert, T., Rudolf, S. & Reinecker, H. (2006). Effectiveness of dialectical behaviour therapy for borderline personality disorder in an inpatient setting. *Behaviour Research and Therapy*, *44*(8), 1211-1217. [see also Fassbinder et al., 2007]

Kröger, C., Harbeck, S., Armbrust, M., & Kliem, S. (2013). Effectiveness, response, and dropout of dialectical behavior therapy for borderline personality disorder in an inpatient setting. *Behaviour Research and Therapy*, *51*(8), 411-416.

Kröger, C., Röepke, S., & Kliem, S. (2014). Reasons for premature termination of dialectical behavior therapy for inpatients with borderline personality disorder. *Behaviour Research and Therapy*, *60*, 46-52.

Kröger, C., Mestel R., Votsmeier-Röhr, A., Kliem, S. (2015). Effectiveness, response, and dropout in psychodynamic therapy for inpatients with borderline personality disorder. An open trial in routine mental healthcare. *Submitted for publication.*

Lana, F., Sánchez-Gil, C., Ferrer, L., López-Patón, N., Litvan, L., Marcos, S., Sierra, A. C., Soldevilla, J. M., Feixas, G., & Pérez, V. (2014). Effectiveness of an integrated treatment for severe personality disorders. A 36-month pragmatic follow-up. *Revista de Psiquiatria y Salud Mental (English Ed.), 8*(1), 3–10.

Laurenssen, E. M. P., Luyten, P., Kikkert, M. J., Westra, D., Peen, J., Soons, M. B. J., van Dam, A.-M., van Broekhuyzen, A. J., Blankers, M., Busschbach, J. J. V., & Dekker, J. J. M. (2018). Day hospital mentalization-based treatment v. specialist treatment as usual in patients with borderline personality disorder: randomized controlled trial. *Psychological Medicine, 48*(15), 2522–2529.

Leerer, C. G. (1997). Outcomes of inpatient cognitive-behavioral treatment of borderline personality disorder. *Dissertation Abstracts International B: Science and, Engineering*.

Leppänen, V., Hakko, H., Sintonen, H., & Lindeman, S. (2016). Comparing effectiveness of treatments for borderline personality disorder in communal mental health care: The Oulu BPD study. *Community Mental Health Journal, 52*(2), 216–227.

Lin, T.-J., Ko, H.-C., Wu, J. Y.-W., Oei, T. P., Lane, H.-Y., & Chen, C.-H. (2019). The effectiveness of dialectical behavior therapy skills training group vs. cognitive therapy group on reducing depression and suicide attempts for borderline personality disorder in Taiwan. *Archives of Suicide Research, 23*(1), 82–99.

Linehan, M. M., Armstrong, H. E., Suarez, A., Allmon, D., & Heard, H. L. (1991). Cognitive-behavioral treatment of chronically parasuicidal borderline patients. *Archives of general psychiatry*, *48*(12), 1060-1064.

Linehan, M. M., Comtois, K. A., Murray, A. M., Brown, M. Z., Gallop, R. J., Heard, H. L., Korslund, K.E., Tutek, D.A., Reynolds, S.K. & Lindenboim, N. (2006). Two-year randomized controlled trial and follow-up of dialectical behavior therapy vs therapy by experts for suicidal behaviors and borderline personality disorder. *Archives of general psychiatry*,*63*(7), 757-766.

Linehan, M. M., McDavid, J. D., Brown, M. Z., Sayrs, J. H., & Gallop, R. J. (2008). Olanzapine plus dialectical behavior therapy for women with high irritability who meet criteria for borderline personality disorder: a double-blind, placebo-controlled pilot study. *Journal of Clinical Psychiatry*, *69*(6), 999-1005.

Linehan, M. M., Korslund, K. E., Harned, M. S., Gallop, R. J., Lungu, A., Neacsiu, A. D., McDavid, J., Comtois, K.A. & Murray-Gregory, A. M. (2015). Dialectical behavior therapy for high suicide risk in individuals with borderline personality disorder: a randomized clinical trial and component analysis. *JAMA Psychiatry*, *72*(5), 475-482.

Löf, J., Clinton, D., Kaldo, V., & Rydén, G. (2018). Symptom, alexithymia and self-image outcomes of mentalisation-based treatment for borderline personality disorder: A naturalistic study. *BMC Psychiatry, 18*(1), 185–185.

Löffler-Stastka, H., Ponocny-Seliger, E., Meißel, T., & Springer-Kremser, M. (2006). Gender aspects in the planning of psychotherapy for borderline personality disorder. *Wiener klinische Wochenschrift*, *118*(5-6), 160-169.

Lyng, J., Swales, M. A., Hastings, R. P., Millar, T., & Duffy, D. J. (2020). Outcomes for 18 to 25-year-olds with borderline personality disorder in a dedicated young adult only DBT programme compared to a general adult DBT programme for all ages 18+. *Early Intervention in Psychiatry, 14*(1), 61–68.

Majdara, E., Rahimian-Boogar, I., Talepasand, S., & Gregory, R. J. (2021). Dynamic deconstructive psychotherapy in Iran: A randomized controlled trial with follow-up for borderline personality disorder. *Psychoanalytic Psychology, 38*(4), 328–335.

Markowitz, J. C., Bleiberg, K., Pessin, H., & Skodol, A. E. (2007). Adapting interpersonal psychotherapy for borderline personality disorder. *Journal of Mental Health*, *16*(1), 103-116.

McMain, S. F., Links, P. S., Gnam, W. H., Guimond, T., Cardish, R. J., Korman, L., & Streiner, D. L. (2009). A randomized trial of dialectical behavior therapy versus general psychiatric management for borderline personality disorder. *The American Journal of Psychiatry*, *166*(12), 1365-1374.

McMain, S. F., Guimond, T., Barnhart, R., Habinski, L., & Streiner, D. L. (2017). A randomized trial of brief dialectical behaviour therapy skills training in suicidal patients suffering from borderline disorder. *Acta Psychiatrica Scandinavica, 135*(2), 138–148.

Meares, R., Stevenson, J., & Comerford, A. (1999). Psychotherapy with borderline patients: I. A comparison between treated and untreated cohorts. *Australian and New Zealand Journal of Psychiatry*, *33*(4), 467-472.

Moen, R., Freitag, M., Miller, M., Lee, S., Romine, A., Song, S., Adityanjee, A. & Schulz, S. C. (2012). Efficacy of extended-release divalproex combined with "condensed" dialectical behavior therapy for individuals with borderline personality disorder. *Ann Clin Psychiatry*, *24*(4), 255-60.

Morey, L. C., Lowmaster, S. E., & Hopwood, C. J. (2010). A pilot study of manual-assisted cognitive therapy with a therapeutic assessment augmentation for borderline personality disorder. *Psychiatry research*, *178*(3), 531-535.

Nadort, M., Arntz, A., Smit, J. H., Giesen-Bloo, J., Eikelenboom, M., Spinhoven, P., van Asselt., Wensing, M. & van Dyck, R. (2009). Implementation of outpatient schema therapy for borderline personality disorder with versus without crisis support by the therapist outside office hours: A randomized trial. *Behaviour Research and Therapy*, *47*(11), 961-973.

Nordahl, H. M., & Nysæter, T. E. (2005). Schema therapy for patients with borderline personality disorder: a single case series. *Journal of Behavior Therapy and Experimental Psychiatry*, *36*(3), 254-264.

Nordahl, H. M., & Wells, A. (2019). Metacognitive therapy of early traumatized patients with borderline personality disorder: A phase-II baseline controlled trial. *Frontiers in Psychology, 10*, 1694–1694.

Nysæter, T. E., Nordahl, H. M., & Havik, O. E. (2010). A preliminary study of the naturalistic course of non-manualized psychotherapy for outpatients with borderline personality disorder: Patient characteristics, attrition and outcome. *Nordic Journal of Psychiatry, 64(2),* 87-93.

Paret, C., Zopfs, M., Keynan, J., Jindrová, M., Stirner, M., Baumeister, S., Aggensteiner, P., Brandeis, D., Hendler, T. & Schmahl, C. (2021). EFP-neurofeedback in adolescents with borderline personality disorder: A proof-of-concept trial. *Neuropsychopharmacology, 46*, 314-315.

Pasieczny, N., & Connor, J. (2011). The effectiveness of dialectical behaviour therapy in routine public mental health settings: an Australian controlled trial. *Behaviour Research and therapy*, *49*(1), 4-10.

Prendergast, N., & McCausland, J. (2007). Dialectic behaviour therapy: a 12-month collaborative program in a local community setting. *Behaviour Change*, *24*(01), 25-35.

Priebe, S., Bhatti, N., Barnicot, K., Bremner, S., Gaglia, A., Katsakou, C., Molosankwe. I., McCrone, P. & Zinkler, M. (2012). Effectiveness and cost-effectiveness of dialectical behaviour therapy for self-harming patients with personality disorder: A pragmatic randomised controlled trial. *Psychotherapy and psychosomatics*,*81*(6), 356-365.

Reiss, N., Lieb, K., Arntz, A., Shaw, I. A., & Farrell, J. (2014). Responding to the treatment challenge of patients with severe BPD: Results of three pilot studies of inpatient schema therapy. *Behavioural and cognitive psychotherapy*,*42*(03), 355-367.

Rizvi, S. L., Hughes, C. D., Hittman, A. D., & Vieira Oliveira, P. (2017). Can trainees effectively deliver dialectical behavior therapy for individuals with borderline personality disorder? Outcomes from a training clinic*. Journal of Clinical Psychology, 73*(12), 1599–1611.

Rüsch, N., Schiel, S., Corrigan, P. W., Leihener, F., Jacob, G. A., Olschewski, M., Lieb. K. & Bohus, M. (2008). Predictors of dropout from inpatient dialectical behavior therapy among women with borderline personality disorder. *Journal of Behavior Therapy and Experimental Psychiatry*, *39*(4), 497-503.

Ryle, A., & Golynkina, K. (2000). Effectiveness of time-limited cognitive analytic therapy of borderline personality disorder: Factors associated with outcome. *British Journal of Medical Psychology*, *73*(2), 197-210.

Sachdeva, S., Goldman, G., Mustata, G., Deranja, E., & Gregory, R. J. (2013). Naturalistic Outcomes of Evidence-Based Therapies for Borderline Personality Disorder at a University Clinic A Quasi-Randomized Trial. *Journal of the American Psychoanalytic Association, 61*(3), 578-584. [see also Gregory & Sachdeva., 2016]

Sachse, S., Keville, S., & Feigenbaum, J. (2011). A feasibility study of mindfulness-based cognitive therapy for individuals with borderline personality disorder. *Psychology and Psychotherapy: Theory, Research and Practice*,*84*(2), 184-200.

Sandell, R., Alfredsson, E., Berg, M., Crafoord, K., Lagerlöf, A., Arkel, I., Crohn, T., Rasch, B. & Rugolska, A. (1993). Clinical significance of outcome in long-term follow-up of borderline patients at a day hospital. *Acta Psychiatrica Scandinavica*, *87*(6), 405-413.

Simpson, E. B., Yen, S., Costello, E., Rosen, K., Begin, A., Pistorello, J., & Pearlstein, T. (2004). Combined dialectical behavior therapy and fluoxetine in the treatment of borderline personality disorder. *Journal of Clinical Psychiatry*, *65(3),* 379-385.

Sinnaeve, R., van den Bosch, L. M. C., Hakkaart-van Roijen, L., & Vansteelandt, K. (2018). Effectiveness of step-down versus outpatient dialectical behaviour therapy for patients with severe levels of borderline personality disorder: A pragmatic randomized controlled trial. *Borderline Personality Disorder and Emotion Dysregulation, 5*(1), 12–12.

Smits, M. L., Feenstra, Di. J., Eeren, H. V., Bales, D. L., Laurenssen, E. M. ., Blankers, M., Soons, M. B. ., Dekker, J. J. ., Lucas, Z., Verheul, R., & Luyten, P. (2020). Day hospital versus intensive out-patient mentalisation-based treatment for borderline personality disorder: Multicentre randomised clinical trial. *British Journal of Psychiatry, 216*(2), 79–84.

Soler, J., Pascual, J. C., Campins, J., Barrachina, J., Puigdemont, D., Alvarez, E., & Pérez, V. (2005). Double-blind, placebo-controlled study of dialectical behavior therapy plus olanzapine for borderline personality disorder. *American Journal of Psychiatry 162(6),* 1221-1224.

Soler, J., Pascual, J. C., Tiana, T., Cebrià, A., Barrachina, J., Campins, M. J., Gich, I., Alvarez. E. & Pérez, V. (2009). Dialectical behaviour therapy skills training compared to standard group therapy in borderline personality disorder: a 3-month randomised controlled clinical trial. *Behaviour Research and Therapy*, *47*(5), 353-358.

Soler, J., Valdepérez, A., Feliu-Soler, A., Pascual, J. C., Portella, M. J., Martín-Blanco, A., ... & Pérez, V. (2012). Effects of the dialectical behavioral therapy-mindfulness module on attention in patients with borderline personality disorder. *Behaviour Research and Therapy, 50(2)*, 150-157.

Sollberger, D., Gremaud-Heitz, D., Riemenschneider, A., Agarwalla, P., Benecke, C., Schwald, O., ... & Dammann, G. (2015). Change in identity diffusion and psychopathology in a specialized inpatient treatment for borderline personality disorder. *Clinical Psychology & Psychotherapy, 22(6),* 559-569.

Stanley, B., Brodsky, B., Nelson, J. D., & Dulit, R. (2007). Brief dialectical behavior therapy (DBT-B) for suicidal behavior and non-suicidal self injury.*Archives of Suicide Research*, *11*(4), 337-341.

Stiglmayr, C., Stecher-Mohr, J., Wagner, T., Meiβner, J., Spretz, D., Steffens, C., Roepke, S., Fydrich, T., Salbach-Andrae, H., Schulze, J. & Renneberg, B. (2014). Effectiveness of dialectic behavioral therapy in routine outpatient care: the Berlin Borderline Study. *Borderline Personality Disorder and Emotion Dysregulation*, *1*(1), 1.

Thormahlen, B., Weinryb, R. M., Norén, K., Vinnars, B., Bagedahl-Strindlund, M., & Barber, J. P. (2003). Patient factors predicting dropout from supportive-expressive psychotherapy for patients with personality disorders. *Psychotherapy Research, 13(4),* 493-509.

Turner, R. M. (2000). Naturalistic evaluation of dialectical behavior therapy-oriented treatment for borderline personality disorder. *Cognitive and Behavioral Practice*, *7*(4), 413-419.

Vaglum, P., Friis, S., Irion, T., Johns, S., Karterud, S., Larsen, F., & Vaglum, S. (1990). Treatment response of severe and nonsevere personality disorders in a therapeutic community day unit. *Journal of Personality Disorders*, *4*(2), 161.

Vaillancourt, K. E. (2012). *Outpatient dialectical behavior therapy at a community mental health center: Outcome study.* [Doctoral dissertation, Antioch University]. OhioLINK Electronic Theses and Dissertations Center.

van den Bosch, L.M.C., Sinnaeve, R. & Nijs, M. (2013). Kortdurende klinische dialectische gedragstherapie voor borderlinepersoonlijkheidsstoornis: Ontwerp van een programma en resultaten pilotstudie. *Tijdschrift voor Psychiatrie,* 55, 165-175.

Verheul, R., van den Bosch, L. M., Koeter, M. W., De Ridder, M. A., Stijnen, T., & Van Den Brink, W. (2003). Dialectical behaviour therapy for women with borderline personality disorder. *The British Journal of Psychiatry*, *182*(2), 135-140.

Walton, C. J., Bendit, N., Baker, A. L., Carter, G. L., & Lewin, T. J. (2020). A randomised trial of dialectical behaviour therapy and the conversational model for the treatment of borderline personality disorder with recent suicidal and/or non-suicidal self-injury: An effectiveness study in an Australian public mental health service. *Australian and New Zealand Journal of Psychiatry, 54*(10), 1020–1034.

Wilberg, T., Friis, S., Karterud, S., Mehlum, L., Urnes, Ø., & Vaglum, P. (1998a). Outpatient group psychotherapy: A valuable continuation treatment for patients with borderline personality disorder treated in a day hospital?: A 3-year follow-up study. *Nordic Journal of Psychiatry*, *52*(3), 213-221.

Wilberg, T., Karterud, S., Urnes, Ø., Pedersen, G., & Friis, S. (1998b). Outcomes of poorly functioning patients with personality disorders in a day treatment program. *Psychiatric Services, 49 (11),* 1462-1467.

Wildgoose, A., Clarke, S., & Waller, G. (2001). Treating personality fragmentation and dissociation in borderline personality disorder: a pilot study of the impact of cognitive analytic therapy. *British Journal of Medical Psychology*, *74*(1), 47-55.

Yeomans, F., Selzer, M., & Clarkin, J. (1993). Studying the treatment contract in intensive psychotherapy with borderline patients. *Psychiatry*, *56*(3), 254-263.

Zinkler, M., Gaglia, A., Arokiadass, S. R., & Farhy, E. (2007). Dialectical behaviour treatment: implementation and outcomes. *The Psychiatrist*, *31*(7), 249-252.

First Author Year (Study Nr.)	Treatment Arm	N	Proportion Dropout (year 1)	Dropout per Quarter (Quarter 1-12)												Dropout per Quarter Available?[1]	Correspondence with author[2]
				1	*2*	*3*	*4*	*5*	*6*	*7*	*8*	*9*	*10*	*11*	*12*		
**Alavi 2021 (571)**	DBTmin	55	0.45	25												Yes	
**Amianto 2011 (7)**	TAU	17	0.06	0	1	0	0									Yes	
	Psychodynamic	18	0.06	0	1	0	0									Yes	
**Arntz 2022 (611)**	ST indiv & group	123	0.19	9	7	1	6	6	2	0	1					Yes	Data provided
	ST group	125	0.28	16	12	4	3	4	1	3	4					Yes	Data provided
	SpecOther (Optimal TAU)	246	0.27	30	16	13	7	10	4	7	8					Yes	Data provided
**Bales 2012 (9)**	MBT	45	0.11	0	1	1	3	2	0							Yes	Data provided
**Barnicot 2019 (502)**	DBT	58	0.59	17	6	3	8									Yes[11]	Data provided
	MBT	32	0.28	1	6	1	1									Yes	Data provided
**Bateman 1999 (13)**	MBT	22	0.14	2	1	0	0	0	0							Yes	
	TAU	22	0.14	3	0	0	0	0	0							Yes	
**Bateman 2009 (17)[3]**	MBT	71	0.27	3	3	7	6	?[3]	?[3]							Partially[3]	Data not provided
	SpecOther (SCM)	63	0.25	5	5	3	3	?[3]	?[3]							Partially[3]	Data not provided
**Bellino 2010 (20)**	SpecOther (IPT)	27	0.19	5	0	0										Yes	
**Ben-Porath 2004 (24)**	DBT	26	0.12	3	0											Yes	Data provided
**Blum 2008 (251)**	TAU	72	0.39	18	10											Partially[4]	Data not provided
**Bohus 2000 (37)**	DBT	24	0	0												Yes	
**Borschmann 2013 (269)**	TAU	42	0.12	3	2											No	Data not provided
**Bos 2010 (252)**	CBT (STEPPS indiv & group)	45	0.36	10	6											Partially	Data not provided
	TAU	38	0.11	2	2											Partially	Data not provided
**Bozatello 2020 (584)**	SpecOther (IPT)	22	0.14	3	0	0										Yes	
	TAU	21	0.19	4	0	0										Yes	
**Brassington 2006 (42)**	DBT	11	0.09	1	0											Yes	
**Brown 2004 (43)**	CBT	32	0.13	2	1	1	0									Partially	No response
**Carmona I Farres 2019 (572)**	DBTmin (Interpersonal Skills)	32	0.25	8												Yes	
	DBTmin (Mindfulness)	33	0.33	11												Yes	
**Carter 2010 (45)**	DBT	38	0.47	12	6											Yes	
	TAU + WL	35	0.11	4	0											Yes	
**Chalker 2015 (423)**	DBT	63	0.57	17	10	5	4									No	Data not provided
**Chanen 2021 (565)**	Mixed (CAT + HYPE)	24	0.37	3	4	3	0	0								Yes	Data for 18+ provided

	Mixed (HYPE+ befriending)	27	0.70	7	7	5	0	0								Yes	Data for 18+ provided
	Mixed (YMHS + befriending)	25	0.88	16	5	1	0	0								Yes	Data for 18+ provided
**Clarkin 2001 (52)**	TFP	23	0.26	2	2	2	0									Yes	
**Clarkin 2007 (53)**	TFP	31	0.29	4	2	2	1?[6]									No	No response
	DBT	30	0.50	7	4	2	2?[6]									No	No response
	Psychodynamic	29	0.28	3	2	2	1?[6]									No	No response
**Comtois 2007 (55)**	DBT	38	0.24	7	2	0	0									Partially[7]	Data not provided
**Comtois 2010 (51)**	DBT	85	0.46	18	10	7	4									No	Data not provided
**Cottraux 2009**	CBT	33	0.39	4	3	3	3									Partially	Data not provided
**(57)**	SpecOther (CCT)	32	0.44	4	3	4	3									Partially	Data not provided
**Davidson 2006 (61)**	CBT (+ TAU)	54	0.06	3	0	0	0									Partially	Data not provided
	TAU	52	0.06	2	1	0	0									Partially	Data not provided
**Del Pozo 2018 (405)**	Mixed	269	0.22	59												Yes	
**Dickhaut 2014 (220)**	ST	18	0.33	4	2	0	0	0	0	0	1	0	0			Yes	Data provided
**Doering 2010**	TFP	52	0.38	15	4	1	0									Yes	
**(67)**	CTBE	52	0.65	28	5	0	1									Yes	
**Farrell 2009 (70)**	ST (+ TAU)	16	0	0	0	0										Yes	
	TAU	16	0.25	2	1	1										No	Data not provided
**Fassbinder 2016 (413)**	ST	10	0.10	0	1	0	0									Yes	
**Feigenbaum 2012**	DBT	25	0.56	5	6	3	0									Partially[8]	No response
**(73)**	TAU	16	0.06	1	0	0	0									Yes	
**Fitzpatrick 2020 (553)**	DBT	101	0.23	6	17											Yes	
**Friedrich 2003 (223)**	DBT	33	0.12	2	0	1	1									Yes	
**Gaglia 2013 (297) (participants doubling Priebe 2014 excluded)**	DBT	62	0.66	24	7	9	1									Yes	Data provided
**Giesen-Bloo 2006**	ST	45	0.07	0	0	2	1	2	0	3	0	0	2	2	0	Yes	
**(75)**	TFP	43	0.37	8	3	1	4	0	0	4	0	1	1	0	0	Yes	
**Gratz 2006 (82)**	Mixed	36	0.28	10												Yes	
**Harley 2007 (89)**	DBT	10	0.50	2	2	1										No	Data not provided
	DBTmin (+TAU)	39	0.59	13	7	3										No	Data not provided
**Hilden 2020 (569)**	ST (+TAU)	25	0.08	1	1											No	Data not provided
	TAU	12	0	0	0											Yes	
**Höschel 2006 (95)**	DBT	26	0.08	2												Yes	

**Jacob 2018 (444)**	ST	14	0.36	2	1	2	0					Yes	Data provided
**Jimenez 2022 (628)**	DBTmin	229	0.45	68	36							No	Data not provided
**Jorgensen 2012 (99)**	MBT	58	0.17	0	3	5	2	0	3	2	1	Yes	Data provided
	Psychodynamic	27	0.11	1	1	1	0	3	0	0	0	Yes	Data provided
**Karterud 1992 (105)**	SpecOther (Therapeutic Community)	34	0.44	8	4	3						No	
**Karterud 2003 (104)**	Mixed	275	0.36	62	36							No	No response
**Kellett 2013 (281)**	SpecOther (CAT)	19	0.05	1	0							Yes	
**Kells 2020 (561)**	DBTmin	41	0.59	11	13							Yes	Data provided
**Kleindienst 2011 (38) (also Bohus 2004)**	DBT	74	0.23	17								Yes	
	TAU + WL	20	0.05	1								Yes	
**Koons 2001 (111)**	DBT	14	0.29	4	0							Yes	
	TAU	14	0.29	4	0							Yes	
**Kramer 2016 (432)**	DBTmin	21	0.27	4	2							Partially	Data provided
	TAU	20	0.29	4	2							Partially	Data provided
**Kröger 2006 (118)**	DBT	50	0.12	6								Yes	
**Kröger 2013 (284)**	DBT	1423	0.10	148								Yes	
**Kröger 2014 (226)**	DBT	541	0.33	176								Yes	
**Kröger 2015 (232)**	Psychodynamic	269	0.28	74								Yes	
**Lana 2015 (415)**	Mixed	40	0.33	8	5							Yes	Data provided
**Laurenssen 2018 (412)**	MBT	54	0.37	8	5	4	3	1	0			Partially[9]	Data partially provided
	TAU	41	0.61	15	7	2	1	1	1			Partially[9]	Data partially provided
**Leerer 1997 (121)**	DBTmin	14	0.29	2	0	2	0					Yes	
**Leppänen 2016 (422)**	Mixed	24	0.17	2	1	1	0					Yes	Data provided
	TAU	47	0.32	5	5	1	4					Yes	Data provided
**Lin 2019 (603)**	DBTmin	42	0.21	9								Yes	
	CBT (group)	40	0.30	12								Yes	
**Linehan 1991 (126)**	DBT	24	0.17	2	1	1	0					Yes	
	TAU	22	0.50	4	3	3	1					Yes	
**Linehan 2006 (130)**	DBT	52	0.19	3	2	3	2					Yes	Data provided
	CTBE	49	0.43	8	7	3	3					Yes	Data provided
**Linehan 2008 (129)**	DBT	24	0.33	5	3							No	Data not provided
**Linehan 2015 (228)**	DBT	33	0.24	3	1	2	2					Yes	Data provided
	DBTmin (individual)	33	0.48	4	5	4	3					Yes	Data provided
	DBTmin (group)	33	0.39	5	3	3	2					Yes	Data provided
**Löf 2018 (411)**	MBT	75	0.19	3	1	4	6	5	4			Yes	Data provided
**Löffler-Stastka 2006 (135)**	Psychodynamic	20	0.80	16								Yes	
**Lyng 2019 (520)**	DBTyouth	24	0.21	2	1	1	1					No	No response

	DBT	13	0.15	1	1	0	0									No	No response
**Majdara 2021 (564)**	SpecOther (DDP)	15	0.13	0	1	1	0									Yes	
	TAU (EUC)	15	0.20	0	2	1	0									Yes	
**Markowitz 2007 (138)**	SpecOther (IPT)	11	0.27	3	0	0										Yes	Data provided
**McMain 2009 (143)**	DBT	90	0.39	12	8	11	4									Yes	Data provided
	SpecOther (GPM)	90	0.38	12	10	5	7									Yes	Data provided
**McMain 2017 (408)**	DBTmin	42	0.31	9	4											Yes	Data provided
**Meares 1999 (147)**	Psychodynamic	48	0.17	3	2	2	1									No	
**Moen 2012 (150)**	DBT	18	0.44	8												Yes	
**Morey 2010 (151)**	CBT (MACT)	8	0.75	6												Yes	
	CBT (MACT+TA)	8	0.88	7												Yes	
**Nadort 2009 (153)**	ST	62	0.06	2	1	0	1	0	3							Yes	
**Nordahl 2005 (157)**	ST	6	0	0	0	0	0	0	0	0	0	0	0	0	0	Yes	
**Nordahl 2019 (518)**	CBT	12	0	0	0	0	0									Yes	
**Nyseater 2010 (158)**	Psychodynamic	32	0.22	4	2	0	1	1	0	0	1					Yes	Data provided
**Paret 2021 (575)**	DBT	22	0.27	6												Yes	
**Pasieczny 2011**	DBT	43	0.07	3	0											Yes	Data provided
**(163)**	TAU + WL	47	0.13	4	2											Yes	Data provided
**Prendergast 2007 (224)**	DBT	16	0.31	3	2											No	Data not provided
**Priebe 2012 (169)**	DBT	40	0.53	8	4	6	3									Yes	Data provided
	TAU	40	0.00	0	0	0	0									Yes	Data provided
**Reiss 2014 (229)**	ST	42	0.02	1	0											Yes	
**Reiss 2014 (230)**	ST	37	0.03	1												Yes	
**Reiss 2014 (231)**	ST	16	0.06	1												Yes	
**Rizvi 2017 (407)**	DBT	50	0.32	7	9											Yes	Data provided
**Rüsch 2008 (173)**	DBT	60	0.32	19												Yes	
**Ryle 2000 (174)**	SpecOther (CAT)	37	0.22	5	3											No	
**Sachdeva 2013**	DBT	25	0.64	6	7	3	0									Yes	Data provided
**(292) (also Gregory**	SpecOther (DDP)	27	0.33	4	3	2	0									Yes	Data provided
**2016)**	TAU	16	0.69	2	4	3	2									Yes	Data provided
**Sachse 2011 (176)**	CBT (MBCT)	26	0.54	14												Yes	
**Sandell 1993 (178)**	Psychodynamic	132	0.27	22	14											No	
**Simpson 2004 (182)**	DBT	25	0.08[5]	2												Yes	
**Sinnaeve 2018**	DBT(outpatient)	42	0.60	23	2	0	0									Yes	Data provided
**(410)**	DBT(inpatient)	42	0.36	8	5	2										Yes	Data provided
**Smits 2019 (400)**	MBT(outpatient)	44	0.05	0	0	1	1	2	1							Yes	Data provided
	MBT(day treatment)	47	0.15	0	1	3	3	1	1							Yes	Data provided
**Soler 2005 (185)**	DBTmin	60	0.30	18												Yes	

**Soler 2009 (186)**	DBTmin	30	0.37	11								Yes	
	TAU	30	0.63	19								Yes	
**Soler 2012 (181)**	TAU	19	0.37	7								Yes	
**Sollberger 2014 (233)**	Mixed	34	0.03	1								Yes	
	TAU	21	0.10	2								Yes	
**Stanley 2007 (189)**	DBT	20	0.05	1	0							Yes	
**Stiglmayr 2014 (222)**	DBT	78	0.29	10	6	4	3					No	Data not provided
**Thormahlen 2003 (299)**	Psychodynamic	12	0.50	4	1	1	0					Yes	
**Turner 2000 (196)**	DBTmin	12	0.25	1	1	1	0					No	No response
	SpecOther (CCT)	12	0.50	3	1	1	1					No	No response
**Vaglum 1990 (218)**	SpecOther (Therapeutic Community)	44	0.32	9	5							No	
**Vaillancourt 2012 (430)**	DBT	241	0.85[10]	123	48	22	12					No	Data not provided
**Van den Bosch 2013 (202)**	DBT	39	0.33	13								Yes	
**Verheul 2003 (206)**	DBT	31	0.45	6	4	4	0					Yes	Data provided
	TAU	33	0.79	23	1	2	0					Yes	Data provided
**Walton 2020 (540)**	DBT	83	0.45	9	12	14	2	1				Yes	Data provided
	Psychodynamic	83	0.42	12	10	8	5	0				Yes	Data provided
**Wilberg 1998a (210)**	Psychodynamic	53	0.47	13	7	5						No	
**Wilberg 1998b (219)**	Mixed	70	0.36	16	9							No	
**Wildgoose 2001 (211)**	SpecOther (CAT)	5	0	0	0							Yes	
**Yeomans 1993 (213)**	TFP	14	0.64	5	2	1	1	0	0	0	0	No	
**Zinkler 2007 (215)**	DBT	49	0.63	14	12	3	2					Yes	Data provided

Note. If dropout per quarter was not available, the data in the table are based on an estimation (see Appendix E). If study duration did not equate a full number of quarters, the dropout in the last quarter was estimated on the basis of the same model (Appendix E) .

[1]Partially means that the dropout numbers per quarter were partially provided in the publication.
[2]For publications published after 2000, authors were emailed with the request to provide treatment dropout per quarter if these numbers were not reported, or only partially reported (e.g., per 6 months), in the publication. Authors either responded with the requested data ("Data provided"), or told that they could not provide the data ("Data not provided") , which could have various reasons (e.g., not documented, data lost, no financial means to look up in the files, demanding a remuneration (financial, co-authorship), or did not respond ("No response").
[3]The publication did not report retention data over this period. Over the first year, dropout per half year was reported though with an idiosyncratic definition ("Early (before 6 months) "dropouts" attended less than a quarter of sessions and late dropouts attended less than half available session

over approximately a year of treatment, the remainder are considered completers."; Bateman & Fonagy (2009), p.1356). The numbers per quarter reported in the table are an approximation.

[4] Thirteen patients did not start TAU thus their dropout time is known.

[5] Three patients that dropped out because of not tolerating the drug washout at start of treatment are not counted as DBT dropouts.

[6] The publication reported numbers that continued into the 9-12 months period (of the study), no dropout during the last quarter was reported. Estimated numbers for Q4 are given in the table.

[7] Reported 9 dropouts in months 0-4; no dropouts after 4 months.

[8] Reported 5 dropouts in quarter 1; 9 dropouts in months 4-9.

[9] The dropout numbers per quarter were partially estimated, as dropout was only reported per 6 months.

[10] Study reported on a subsample of participants who received and completed at least one year of DBT treatment.

[11] For one participant, the time of dropout was unknown. This was estimated to be in quarter 3.

Appendix E. Estimation of survival per quarter in studies not reporting enough details

1. Estimating the basic time model of survival chance

Estimation of survival per quarter was only necessary for studies not reporting dropout in enough detail to reconstruct survival rates per quarter. We based the estimation model on the studies with complete data, by running a GLMM survival analysis with only Quarter in the fixed part and study as random intercept. The estimated survival chances per quarter are given in Table E.1. We fitted a logistic function to the values in that table. The best fitting logistic function is $1/(1+\exp(-0.3331844555\times k - 1.387590251))$, with k = the number of the quarter (Figure E.1), resulting in survival chances very near those from the GLMM model, $R^2 = .99989$, Table E.1.

The random intercepts in the GLMM account for deviations of individual studies from the overall pattern in Table E.1. For imputation purposes we need these adjusted values. We describe the adjustments in next.

Table E.1. GLMM survival chance estimates per quarter and estimated survival chances from a logistic model.

Quarter k	Pooled Survival Chance GLMM survival analysis	Estimated Survival Chance $p_k = 1/(1+\exp(-0.3331844555\times k + -1.387590251))$
1	.848	.848
2	.886	.887
3	.916	.916
4	.938	.938

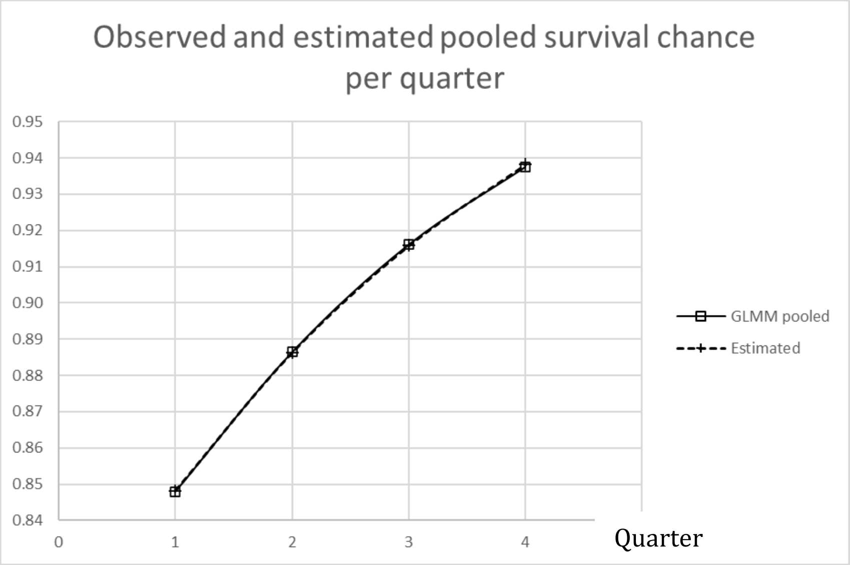

Figure E.1. Survival chances per quarter (quarters 1-4) and approximation by a logistic function.

2. Adjustments of the survival probabilities for individual studies

The interval survival probabilities, averaged across studies, are modeled by

$$\pi_{0k} = 1/(1 + \exp(-0.3331844555 \cdot k - 1.387590251)), \quad k = 1,2,\ldots,Q \qquad (1)$$

where Q is the number of quarters observed. For an individual study these probabilities are somewhat different, but the hazard model underlying their estimate presumes that, as a function of k, they are determined by this underlying common functional form. Their deviation with respect to this common underlying form is modeled in the hazard model by means of a random intercept. It turns out that the relationship between the interval survival probabilities for an individual study and the probabilities defined above is mathematically given by

$$\pi_{ik} = \pi_{0k}^{e^{\gamma_i}}, \quad (2)$$

where γ_i is the study i's random intercept in the hazard model. The individual $\gamma_i's$ are not directly estimated in the multilevel generalized linear model used in this meta-analysis. However, we can derive a method-of-moments estimator for e^{γ_i}. It is given by

$$\widehat{e^{\gamma_i}} = \frac{\ln(N_i - M_i) - \ln N_i}{\sum_{k=1}^{Q_i} \ln \pi_{0k}} \, (3)$$

Here N_i is the number of observations (patients) in study i and M_i is the total number of dropouts in that study. This estimator is guaranteed to be non-negative, and yields a total study survival proportion from the π_{0k} that is equal to the observed total survival proportion $\frac{(N_i - M_i)}{N_i}$.

For studies that have a duration that is not a full quarter, but say, Q_i full quarters plus a fraction r_i of a quarter, the formulas can be adjusted to

$$\pi_{ik} = \begin{cases} \pi_{0k}^{e^{\gamma_i}} & \text{for } k = 1,\ldots,Q_i \\ \pi_{i(Q_i + r_i)} = \left(\pi_{0k}^{r_i}\right)^{e^{\gamma_i}} & \text{for the final observed fractional quarter} \end{cases} \qquad (4)$$

where e^{γ_i} is replaced by the estimate

$$\widehat{e^{\gamma_i}} = \frac{\ln \widehat{S_{T_i}}\,(Q_i + r_i)}{r_i \ln \pi_{0(Q_i+1)} + \sum_{k=1}^{Q_i} \ln \pi_{0k}}, (5)$$

where $\widehat{S_{T_i}}(Q_i + r_i)$ is the proportion of survivors *(N_i – M_i)/N_i* at the end of study *i*. This is based on an approximation of a piecewise constant dropout rate. For the final analyses, survival rates of incomplete quarters are extrapolated to cover the complete quarter, assuming a constant survival rate for that quarter. However, when the fraction is very small (≤ 2 weeks) the incomplete quarter is excluded from the analyses.

3. Multiple imputation

The estimation model outlined above leads to idealized (point) estimates of survival rates per quarter, whereas in reality there will be random fluctuations. To model that, 20 imputed data sets are created, using a binomial distribution around the estimated survival numbers per quarter, under the restriction that the sum of the survival numbers over the quarter equals the observed total survival number (N_i-M_i) / N_i .

4. Mathematical derivation

The probability that a case does not drop out before time Q can be decomposed into a product of survival time interval probabilities:

$$\begin{aligned} P(T > Q) &= P(T > Q|T > Q-1)\cdot P(T > Q|T > Q-1)\cdots P(T > 1|T > 0)P(T > 0) \\ &= P(T > 0)\prod_{q=1}^{Q} P\,(T > q|T > q-1) \end{aligned} \qquad (6)$$

We will assume $P(T > 0) = 1$, and we will write $\pi_{iq} = P(T_i > q \mid T_i > q-1)$ — the survival probability within the interval $(q-1, q]$, given survival up till $q-1$.

The probabilities π_{iq} can be directly estimated from the data as $\widehat{\pi_{iq}} = \frac{N_{i,q-1}-M_{iq}}{N_{i,q-1}}$, where $N_{i,q-1}$ is the number of cases still 'alive' at time $q-1$, and M_{iq} is the number of cases that droped out in the interval $(q-1, q]$. The Kaplan-Meyer estimate of the survival curve is then

$$\hat{S}_i(q) = \prod_{k=1}^{q} \hat{\pi}_{iq}\,, \qquad (7)$$

which is a noisy but unbiased estimate. The survival curve is somewhat difficult to model directly, and that is why often the hazard rate is modeled instead.

In survival analysis the hazard rate is modeled as a function of time t (here in units of *quarter* of the year), and differences between cases are often modeled as proportional (e.g., as in the Cox proportional hazards model):

$$\lambda_i(t) = \frac{f_{T_i}(t)}{1 - F_{T_i}(t)} = \frac{f_{T_i}}{S_{T_i}(t)} = \lambda_0(t)e^{\gamma_i} \qquad (8)$$

Here T_i is the survival time for case i (= *study* in the dropout meta-analysis), f_{T_i} and F_{T_i} are the density and distribution of T_i respectively, $\lambda_0(t)$ is the hazards curve shape that is common to all the cases (studies), and γ_i is a constant that determines the deviation of the i case from the common $\lambda_0(t)$. The function $S_{T_i}(t)$ is a convient shorthand for $1 - F_{T_i}(t)$, and is called the *survival curve*, because it traces the proportion of an initial sample should be expected to survive over time.

In our meta-analysis, $\lambda_0(t)$ was parameterized as a piecewise constant function,

$$\lambda_0(t) = \begin{cases} \lambda_1 & 0 < t \leq 1 \\ \lambda_2 & 1 < t \leq 2 \\ \lambda_3 & 2 < t \leq 3 \\ \lambda_4 & 3 < t \leq 4 \end{cases}, \qquad (9)$$

in order to make as few assumptions about the shape of the curve. But in the following we won't make *any* assumption regarding $\lambda_0(t)$. The γ_i's were modeled as random effects $\gamma_i \sim N(0, \sigma_\gamma^2)$, for which only the variance σ_γ^2 was estimated in the model.

By definition of the hazard rate, we have to following relation:

$$\lambda_i(t) = -\frac{d}{dt}\ln S_{T_i}(t) \Leftrightarrow S_{T_i}(t) = \exp\left(-\int_0^t \lambda_i(s)ds\right).$$

The integral in the exponent implies that the survival function factors over time

$$\begin{aligned} S_{T_i}(t + \Delta t) &= \exp\left(-\int_0^{t+\Delta t} \lambda_i(s)ds\right) = \exp\left(-\int_0^t \lambda_i(s)ds - \int_t^{t+\Delta t} \lambda_i(s)ds\right) \\ &= S_{T_i}(t)\exp\left(-\int_t^{t+\Delta t} \lambda_i(s)ds\right). \end{aligned} \qquad (11)$$

Applying this to the intervals $(0,1], (1,2], \ldots, (q-1, q]$, we have

$$S_{T_i}(q+1) = \prod_{k=1}^{q+1} \pi_{ik} = \prod_{k=1}^{q} \pi_{ik} \cdot \pi_{i(q+1)} = S_{T_i}(q) \cdot \pi_{i(q+1)}. \qquad (12)$$

where $\pi_{ik} = e^{-\int_k^{k+1}\lambda_i(s)ds}$. From $\lambda_i(t) = \lambda_0(t)e^{\gamma_i}$ we have

$$e^{-\int_k^{k+1}\lambda_i(s)ds} = e^{-e^{\gamma_i}\int_k^{k+1}\lambda_0(s)ds} = \left(e^{-\int_t^{t+\Delta t}\lambda_0(s)ds}\right)^{e^{\gamma_i}} = (\pi_{0k})^{e^{\gamma_i}}, \quad \text{for each } k = 1,2,\ldots. \qquad (13)$$

On average $\gamma_i = 0$, in which case $e^0 = 1$. Furthermore, if $Q_i \in \mathbb{N}$ is the last time the survival was observed to be N_{iQ}, then

$$S_{T_i}(Q_i) = \prod_{k=1}^{Q_i} (\pi_{0k})^{e^{\gamma_i}} = \left(\prod_{k=1}^{Q_i} \pi_{0k}\right)^{e^{\gamma_i}}, \qquad (14)$$

and so we may estimate e^{γ_i} given $\pi_{0k}, k = 1, \ldots, Q_i$ from

$$\widehat{e^{\gamma_i}} = \frac{\ln \widehat{S_{T_i}}(Q_i)}{\sum_{k=1}^{Q_i} \ln \pi_{0k}}, (15)$$

or $\hat{\gamma}_i = \ln\left|\ln\hat{S}_{T_i}(Q_i)\right| - \ln\left|\sum_{k=1}^{Q_i} \ln \pi_{0k}\right|$. Here $\hat{S}_{T_i}(Q_i) = \frac{N_{iQ_i}}{N_i} = \frac{N_i - M_i}{N_i}$, where M_i is the total number of dropouts for study i.

Note that for studies of non-integer duration this leads to a natural extension: Say that a study lasted $3\frac{1}{2}$ unit intervals, then

$$S_{T_i}\left(3\frac{1}{2}\right) = S_{T_i}(3)\exp\left(-\int_3^{3\frac{1}{2}} \lambda_i(s)ds\right) = \left(\prod_{k=1}^{3} \pi_{ik}\right)\exp\left(-\int_3^{3\frac{1}{2}} \lambda_i(s)ds\right) \qquad (16)$$

The question is how we can evaluate $\exp\left[\int_3^{3\frac{1}{2}} \lambda_i(s)ds\right]$. If we make our modeling approximation $\lambda_0(t) = \lambda_4$ for $3 < t \leq 4$, then $\exp\left[\int_3^{3\frac{1}{2}} \lambda_i(s)ds\right] = \exp\left[e^{\gamma_i}\int_3^{3\frac{1}{2}} \lambda_4 \; ds\right] = \exp\left[e^{\gamma_i}\frac{1}{2}\lambda_4\right] = \left(e^{\frac{\lambda_4}{2}}\right)^{e^{\gamma_i}} = \left(\pi_{04}^{\frac{1}{2}}\right)^{e^{\gamma_i}}$. Hence,

$$S_{T_i}\left(3\frac{1}{2}\right) = \left(\prod_{k=1}^{3} \pi_{ik}\right)\pi_{i4}^{\frac{1}{2}} = \left[\left(\prod_{k=1}^{3} \pi_{0k}\right)\pi_{04}^{\frac{1}{2}}\right]^{e^{\gamma_i}} \qquad (17)$$

Similarly, for any fraction r of a full interval on top of the q-th full interval, we have

$$S_{T_i}(q + r) = \left[\left(\prod_{k=1}^{q} \pi_{0k}\right)\pi_{0(q+1)}^{r}\right]^{e^{\gamma_i}} \qquad (18)$$

in the approximation that $\lambda_0(t)$ is constant within each interval. For those cases the estimate in equation (15) becomes

$$\widehat{e^{\gamma_i}} = \frac{\ln \widehat{S_{T_i}}\,(Q_i + r_i)}{r_i \ln \pi_{0(Q_i+1)} + \sum_{k=1}^{Q_i} \ln \pi_{0k}},$$

where $\hat{S}_{T_i}(Q_i + r_i) = \frac{(N_i - M_i)}{N_i}$ the proportion of survivors at the end of the study.

Appendix F. Imputation procedure.

Imputation of number of dropouts across quarters was conditioned on the total number of dropouts as observed after the last period. For most studies the last period observed was the fourth quarter, but for a few studies this was two or three quarters.

If dropout probability estimates $\hat{p}k$ per time period Tk are available, $k = 1, \ldots, q$, and a total dropout N after Tq is known, the dropout per Tk can be imputed with

$nk \sim \text{binom}(N - \sum jk\text{-}1\ nj,\ \hat{p}k/\sum j{=}k\ \hat{p}j)$,

where ~ binom(m, p) indicates a binomially distributed random variable that represents the number of 'successes' out of a sequence of m trials with success probability p.

For each study i for which the dropout in each quarter could not be extracted from the published paper or from the authors, estimates $\hat{p}ik$, $k = 1, ..., q$, were obtained for each quarter from a simple regression model. We did not use the model eventually fitted in order to avoid biasing the imputations towards the model. These estimates were then used together with the studies observed total dropout Ni to generate imputed values using the random number generator in SPSS. An SPSS macro is available upon request.

The entire data set was imputed in this way 20 times. For model selection purposes, these 20 data sets were analyzed as if they constituted a single data set, attaching weights to each observation such that each imputed data set contributed only one twentieth to the total number of degrees of freedom. This made it practically feasible to do backward stepdown model selection. Subsequently, the resulting model was then fitted to each of the individual imputed data sets to obtain regression coefficients and difference contrasts, including their standard errors per imputation data set. The resulting sets of 20 regression coefficients and difference contrasts where then combined to obtain a multiple imputation estimate and standard error for each. To this end we used the standard formulas (“Rubin’s rule”) (Donders, et al., 2006; Kim, 2004)

$\hat{\beta}_0 = \sum_r \hat{\beta}_r / M$, and

$V(\hat{\beta}) = \sum_r(\hat{\beta}_r - \hat{\beta}_0)^2 / (M - 1) + (1 + 1/M) * \sum_r SE(\hat{\beta}_r)^2 / M$.

Here $\hat{\beta}r$ is the estimate for the r-th imputed data set ($r = 1, ..., M$, $M = 20$), and $SE(\hat{\beta}r)$ is the estimated standard error for this parameter. Both are obtained from the SPSS output. The imputation estimate $\hat{\beta}0$ is hence, a simple average of the estimates from the imputed data sets. Its standard error is obtained as $SE(\hat{\beta}0) = \sqrt{}(V(\hat{\beta}0))$.

Donders, A. R. T., van der Heijden, G. J., Stijnen, T., & Moons, K. G. (2006). Review: a gentle introduction to imputation of missing values. *Journal of Clinical Epidemiology, 59(10)*, 1087-1091.

Kim, J. K. (2004). Finite sample properties of multiple imputation estimators. *Annals of Statistics, 32,* 766-783.

Appendix G. Funnel plots for the 10 treatment categories (complete data set).

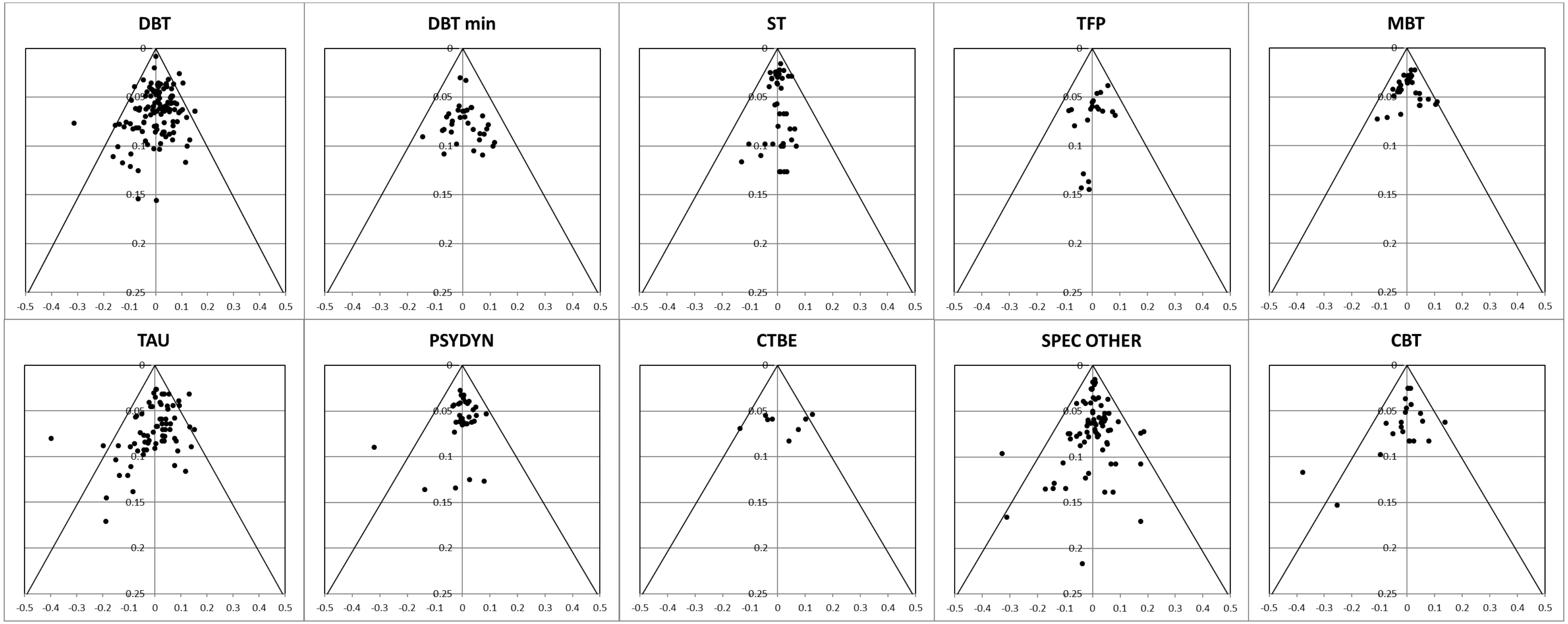

Note. The x-axis is the difference between observed and predicted treatment retention per quarter (per study arm) (expressed as proportion, i.e. between 0 and 1), the y-axis the study precision (the s.e. of the observed treatment retention of the study arm at the pertinent quarter).

Appendix H. Funnel plots for the 10 treatment categories (reduced data set).

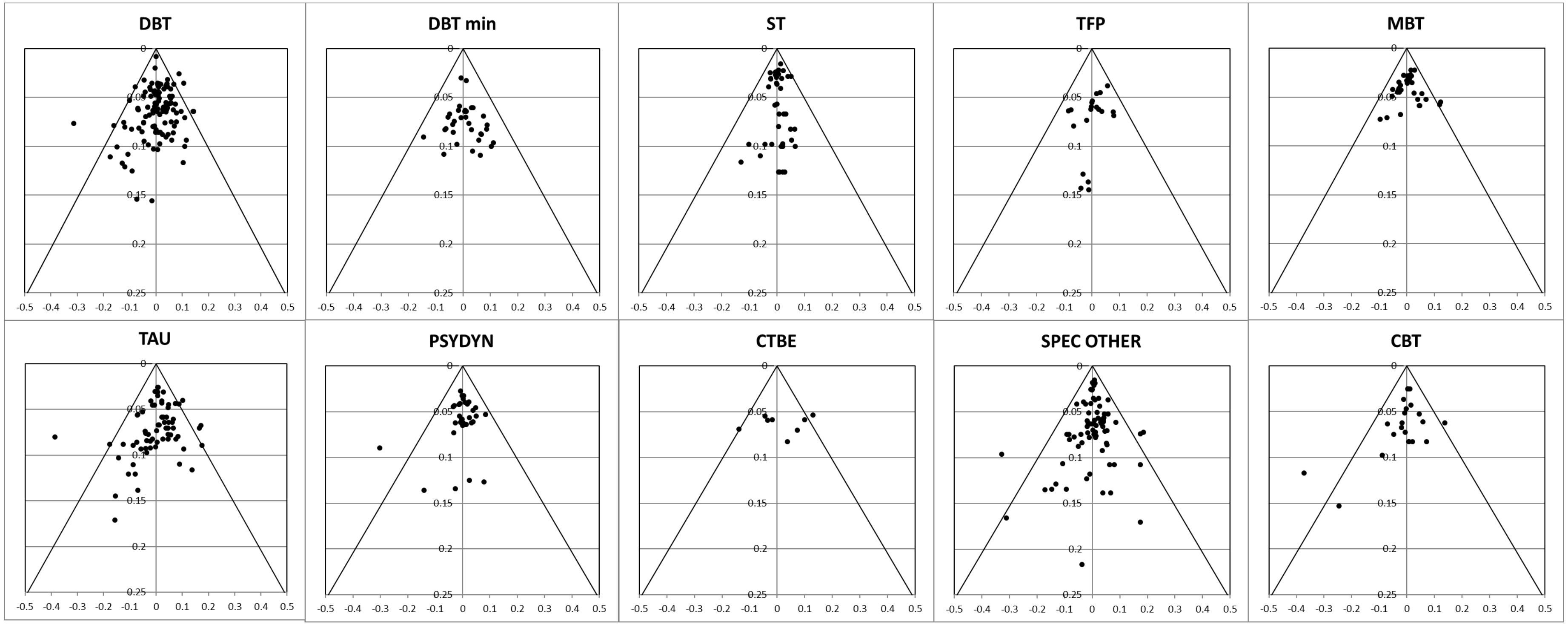

Note. The x-axis is the difference between observed and predicted treatment retention per quarter (per study arm) (expressed as proportion, i.e. between 0 and 1), the y-axis the study precision (the s.e. of the observed treatment retention of the study arm at the pertinent quarter). Data set after deleting the DBT-arms of the Priebe et al. and Gaglia et al. studies.
